# Interaction of the SXT/R391 element ICE*Pmi*Jpn1 with its natural host *Proteus mirabilis*

**DOI:** 10.1128/spectrum.00339-25

**Published:** 2025-05-23

**Authors:** Douglas Lyra de Holanda Fonseca, Gaby Soares Scheunemann, Bruna Nakanishi Fortes, Kelly Ishida, Rodrigo S. Galhardo

**Affiliations:** 1 Department of Microbiology, Institute of Biomedical Sciences, University of São Paulo, São Paulo, Brazil; Universidad Maimonides, Buenos Aires, Argentina

**Keywords:** ICEs, SXT/R391, *Proteus mirabilis*, fitness, swarming, biofilm, persisters, ICE*Pmi*Jpn1, conjugation, MGEs

## Abstract

Integrative and conjugative elements (ICEs) of the SXT/R391 family are mobile genetic elements that integrate into the bacterial host chromosome and can be transferred horizontally, spreading antimicrobial resistance genes. Our study aimed to evaluate aspects of the relationship between ICE*Pmi*Jpn1, one of the most widespread SXT/R391 variants, with its natural host *Proteus mirabilis*. For this investigation, we used isogenic strains (containing or not the ICE*Pmi*Jpn1) that enabled us to evaluate the influence of this element on several physiological aspects of *P. mirabilis* as well as the effect of different *P. mirabilis* genetic backgrounds on the conjugative transmission of the element. ICE*Pmi*Jpn1 did not impact the fitness, self-recognition, swarming, pathogenicity, and persistence abilities of this bacterium but increased biofilm formation in one strain. Additionally, conjugative transfer of the element to *Escherichia coli* is widely variable when different *P. mirabilis* strains are used as donors in mating assays. Our results indicate that ICE*Pmi*Jpn1 has no adverse effects on the physiology or pathogenicity of *P. mirabilis*, reflecting a stable association between this element and its host. Furthermore, the findings support the notion that ICE transfer between bacteria is influenced not only by element-specific regulators but also by strain-specific factors.

**IMPORTANCE:**

Mobile genetic elements play a key role in the spread of antimicrobial resistance, raising concerns about multidrug-resistant bacteria, yet their interactions with bacterial hosts are not well characterized. This study explores the relationship between ICE*Pmi*Jpn1, a globally distributed SXT/R391 integrative and conjugative element (ICE), and its natural host *Proteus mirabilis*, revealing minimal effects on bacterial fitness and pathogenicity. Nevertheless, strain-specific factors significantly influence conjugative transfer. These findings highlight the need for further research on host-dependent regulatory mechanisms that drive the spread of these elements. Understanding these dynamics is essential for developing strategies to mitigate the dissemination of antibiotic resistance in clinically relevant bacterial populations.

## INTRODUCTION

The gram-negative bacillus *Proteus mirabilis* is commonly associated with opportunistic infections in humans (1, 2). It is an *Enterobacterales* of the *Morganellaceae* family (3) that can inhabit both the environment and the gastrointestinal tract of vertebrates (1). In humans, it is capable of infecting wounds, eyes, and the digestive system, but it is primarily involved in urinary infections, particularly associated with clinical hospitalizations and prolonged use of catheters (1, 2). A distinguishing feature of *P. mirabilis* is its rapid swarming motility (2, 4), but it also exhibits several important traits, including biofilm formation (1, 4) and competition with other bacteria through type VI secretion system (T6SS)-mediated toxin/antitoxins modules, which play a role in bacterial recognition (5, 6).

*P. mirabilis* is intrinsically resistant to tetracyclines and polymyxins, but it remains susceptible to β-lactams due to the absence of genes encoding β-lactamases (*bla*) in its chromosome, as well as to other classes of antimicrobials (7, 8). However, despite this natural susceptibility, *P. mirabilis* can survive antimicrobial treatment through bacterial persistence, a widespread phenomenon (9, 10) in which metabolically distinct subpopulations survive lethal doses of antimicrobials, leading to recurrent infections, without the involvement of resistance genes (10, 11). In addition, the acquisition of mobile genetic elements (MGEs) plays a crucial role in the spread of antimicrobial resistance (12, 13). For instance, *ampC* genes confer resistance to most β-lactams, with *CMY-2* being the most globally widespread AmpC variant, primarily transmitted via plasmids or integrative and conjugative elements (ICEs) (7, 14, 15).

Integrative and conjugative elements (ICEs), also known as conjugative transposons, are MGEs that integrate into the bacterial chromosome and regulate their own transfer to a new host. These elements play a crucial role in the horizontal transfer of resistance genes and are widely distributed among bacteria, having been described in environmental, veterinary, and clinical strains (16–18). Research on ICEs is relatively recent, and the term was established in the early 2000s (19) after observations that these elements shared common characteristics, including chromosomal integration site, excision into a circular intermediate, and the presence of bacteriophage- and plasmid-like modules (18, 19).

One of the main families of ICEs is SXT/R391. Initially classified as plasmids, these elements were later reclassified after the observation that the *Vibrio cholerae* SXT element and *Providencia rettgeri* R391 shared high homology and, unlike plasmids, integrate into the host chromosome (16, 20, 21). SXT/R391 ICEs specifically integrate into the 5′ portion of the *prfC* gene, which encodes the peptide releasing factor 3. These large elements range from 79 to over 110 kb and consist of a core set of genes involved in regulation, integration, and transfer. These elements also contain hotspots (HS1-HS5) and variable regions (VRI-VRV) that allow the insertion of genes encoding antimicrobial resistance, heavy metal tolerance, and other adaptive traits (16, 18, 21–23).

ICE transfer is mediated by proteins encoded within the element itself, which provides all the necessary factors for integration and conjugation. Site-specific insertion and excision from the host chromosome are regulated by integrase (*int*), a recombinase, and excisionase (*xis*), in a mechanism similar to that of λ phages. Upon excision, the element forms an extrachromosomal circular molecule that is subsequently transferred as single-stranded DNA (ssDNA) bound to a relaxase, resembling conjugative plasmid transfer. Conjugation occurs via the type IV secretion system (T4SS), which forms a pore during the connection between the donor and recipient cells, enabling ssDNA transfer (16, 18, 19, 22). Once in the recipient cell, integrase catalyzes recombination between the *attP* site on the ICE and the *attB* site in the bacterial chromosome, resulting in chromosomal integration and restoration of a functional *prfC* gene (16, 24, 25).

The regulation of SXT/R391 ICE conjugation involves CroS and SetR, which interact similarly to the λCI and λCro proteins that control phage lytic and lysogenic cycles (26). SetR represses the expression of *setCD* genes, which activate the expression of genes involved in excision, conjugation, and chromosomal integration (16, 17, 26). Under normal conditions, this regulatory network tightly controls ICE transfer. However, in response to genotoxic stress, the SOS response—a bacterial defense mechanism triggered by DNA damage—increases conjugation frequency (27, 28). The SOS response is governed by LexA, which represses SOS genes, and RecA, which detects DNA damage and activates the response (29, 30). In the SXT/R391 family, RecA activation promotes the self-cleavage of SetR, derepressing conjugation-related genes (27).

SXT/R391 ICEs are predominantly found in *Vibrio*, *Proteus,* and related genera (18). However, it remains unclear why these species serve as their primary hosts, given that these elements can be transferred under laboratory conditions to a broad range of γ-Proteobacteria (28, 31). Interestingly, these ICEs persist in bacteria even when they confer no apparent adaptive advantage, including variants lacking antimicrobial resistance genes (18, 23). Furthermore, SXT/R391 ICEs have been shown to impose a fitness cost on *Escherichia coli* (31, 32), and ICE-related genes have been implicated in *E. coli* lysis following UV exposure (33), but not in *P. mirabilis* (28), suggesting co-evolution with specific hosts.

Our research group previously identified SXT/R391 ICEs in five *P. mirabilis* strains from a collection of 76 Brazilian clinical isolates (23). Genome sequencing revealed that one of these ICEs was identical to ICE*Pmi*Jpn1, originally identified in Japan but globally distributed (7, 34–37). This ICE carries a β-lactam resistance gene (*bla*_CMY-2_) and other genes with varied or unknown function within its variable regions and hotspots (23). This study aimed to evaluate whether the presence of ICE*Pmi*Jpn1 could negatively or positively impact aspects of the physiology and pathogenicity of its natural host, *P. mirabilis*. Additionally, we investigated the conjugative transfer ability of different *P. mirabilis* strains under normal conditions and following DNA damage to identify potential variations in transfer patterns within the species.

## MATERIALS AND METHODS

### Bacterial strains

To evaluate the biological effects of ICE*Pmi*Jpn1 carriage by *P. mirabilis*, we used previously obtained pairs of strains consisting of clinical isolates that initially did not have SXT/R391 ICEs and their counterparts that received ICE*Pmi*Jpn1. This element confers resistance to ampicillin (Amp^R^) through the *bla*_CMY-2_ gene and was originally identified in a clinical isolate from Brazil, PmBR19 (23, 28, 38). The selected strains did not present a clonal relationship, based on ERIC-PCR analyses (38), and are shown in Table 1.

**TABLE 1:** List of strains used in this study^*a*^

Strain	Species	Characteristics	Source
PmBR19	*P. mirabilis*	ICE*Pmi*Jpn1	(38)
ATCC25933	*P. mirabilis*	No SXT/R391 ICEs	
PmBR574	*P. mirabilis*	No SXT/R391 ICEs	(38)
PmBR622	*P. mirabilis*	No SXT/R391 ICEs	(38)
PmBR28	*P. mirabilis*	No SXT/R391 ICEs	(38)
PmBR51	*P. mirabilis*	No SXT/R391 ICEs	(38)
RSG832	*P. mirabilis*	ATCC with ICE*Pmi*Jpn1	(28)
RSG833	*P. mirabilis*	PmBR574 with ICE*Pmi*Jpn1	(28)
RSG828	*P. mirabilis*	PmBR622 with ICE*Pmi*Jpn1	(28)
RSG829	*P. mirabilis*	PmBR28 with ICE*Pmi*Jpn1	(28)
RSG830	*P. mirabilis*	PmBR51 with ICE*Pmi*Jpn1	(28)
RSG875	*E. coli*	MG1655 Rif^R^	(28)
RSG1165	*E. coli*	MG1655 Δ*LacZ*::Cm Cm^R^	Beny Spira

^*a*^ Cm^R^, chloramphenicol resistant; Rif^R^, rifampicin resistant.

### Fitness comparison

The comparison of fitness among the isogenic strain pairs was performed by monitoring the growth by turbidity measurements, using optical density at 600 nm (OD_600_), in the following media: Brain Heart Infusion (BHI), Tryptic Soy Broth (TSB), Lysogeny Broth (LB), and *Proteus mirabilis* Minimal Salt Medium (PMSM) [per liter: 10.5 g K_2_HPO_4_, 4.5 g KH_2_PO_4_, 0.47 g sodium citrate, 1 g (NH_4_)_2_SO_4_, and, after autoclaving, 1 mL 1 M MgSO_4_, 10 mL glycerol 20%, and 1 mL of filtered 1% nicotinic acid] (39). Saturated cultures were adjusted to an initial OD_600_ of 0.05, using the spectrophotometer NanoPhotometer C40 (Implen, Munich, Germany), in 35 mL of PMSM and placed in 250 mL Erlenmeyer flasks. Turbidity was measured every 1 hour until culture saturation was established. Alternatively, saturated cultures were adjusted to an initial OD_600_ of 0.05 in 3 mL of the other tested media (BHI, TSB, or LB), and 1 mL was added into each well, in duplicates, in 24-well flat bottom plates. Plates were incubated overnight at 37°C in a Varioskan LUX microplate reader (Thermo Fisher Scientific, Waltham, USA), with OD_600_ readings every 15 minutes.

### Self-recognition experiment (Dienes' lines formation)

Strains bearing the ICE were co-cultured with their isogenic counterpart (without the ICE) or with strain PmBR19, the original donor of ICE*Pmi*Jpn1, to assess whether there was self-recognition or formation of Dienes' lines, demonstrating competition. After overnight growth at 37°C and adjustment to an OD_600_ of 0.1, we inoculated 5 µL of two different cultures at opposite points in LB with 1.5% agar plates and incubated them at 37°C for 18 h. After growth, it was evaluated whether there was a merge or formation of Dienes' lines at the encounter of the swarms from the two different strains.

### Motility assay (swarming)

Swarming motility of the isogenic strains was compared after inoculating 5 µL of a culture adjusted to an OD_600_ of 0.1 at the center of a 140 mm plate with LB and 1.5% agar (39). The migration distance of the colonies from the point of inoculation was measured after 15 hours of incubation at 37°C, with technical triplicate and replicates on different days.

### Biofilm formation assay

The quantification of biofilm production by the isogenic strain pairs was performed using the methodology described by O'Toole (40) using crystal violet (CV) staining in 96-well polystyrene microplates. We inoculated 125 µL of a culture adjusted to an OD_600_ of 0.1 in TSB into each well in eight technical replicates, with biological replicates on different days. After growth for 18 hours at 37°C, the excess culture in the wells was removed, and the microplates were washed in distilled water. After removing excess water, 125 µL of 0.1% CV solution was inserted into the wells and left to act for 10 min. After this period, CV was removed with distilled water, and the microplates were placed to dry overnight at room temperature. Finally, 125 µL of 30% acetic acid was inserted into each well and incubated for 10 min for resuspension of the stained biofilm. This material was transferred to a new 96-well microplate, and the absorbance was measured at OD_550_ through an Epoch 2 microplate reader (BioTek, Vermont, USA).

### Infection assay in *Galleria mellonella*

We used *Galleria mellonella* as an invertebrate model to compare the virulence of the isogenic strains with and without ICE*Pmi*Jpn1, in accordance with the methodology described by Ramarao et al. (41), where bacteria are inoculated in the last left proleg of each larva. The bacteria were cultivated in LB and diluted to a concentration of 10^4^ CFU/mL in phosphate buffer saline (PBS). Larvae of *G. mellonella* were selected in their last larval stage, with 2–2.5 cm in length, each group having 16 specimens. These were disinfected using 70% alcohol before inoculation of 10 µL (approximately 10^2^ CFU) of the bacterial suspension or PBS in its last left proleg using microsyringes (Hamilton, Nevada, USA). The larvae were incubated at 37°C and monitored to assess the mortality caused by the pathogen, with readings taken every two hours after 10 hours of inoculation.

### Antibiotic killing assays for analysis of persister cells

The isogenic strain pairs were tested for susceptibility to the antimicrobials used for persister formation assays (amikacin and ciprofloxacin) by determination of minimal inhibitory concentrations (MICs), following the microdilution method described by Wiegand et al. (42). The assays were conducted in 96-well plates using Mueller Hinton (MH) medium, with initial antimicrobial concentrations based on resistance values recommended by the Clinical and Laboratory Standards Institute (CLSI) (2020) for *Enterobacterales*. The MIC was determined by the well with the lowest concentration of the antimicrobial where there was no visible bacterial growth.

The number of persistent cells was evaluated by counting CFU/mL after exposure to high concentrations of amikacin and ciprofloxacin, according to the methodology described by Liu et al. (11), with modifications. Isogenic strains were cultured overnight in 11 mL of LB to reach the stationary phase, 10 mL of this material was transferred to new tubes, centrifuged, and resuspended in the same amount of 0.9% saline, starting the treatment with the antibiotics at 37°C without agitation. Alternatively, we performed the start of treatment during exponential growth, where saturated cultures had OD_600_ adjusted to 0.05 and incubated at 37°C until they reached an OD_600_ of 0.4–0.5 to start treatment. Prior to antimicrobial exposure, 100 µL of culture was transferred to microtubes containing 900 µL of 0.9% saline, serially diluted, and 10 µL was plated on LB plates to determine initial cell count. At 3, 24, 72, 120, 168 and 192 hours post-treatment, 100 µL of culture was transferred to microtubes containing 900 µL of 0.9% saline, centrifuged, and washed twice to remove residual antimicrobials. After serial dilution, 10 µL of each sample was plated on LB agar without antimicrobials and incubated overnight at 37°C. Viable cell counts were determined by evaluating CFU on the plates and comparing them to the initial cell count prior to treatment, determining the survival fraction.

### Mating assays

Quantitative conjugation experiments to assess the spontaneous and damage-induced transfer of ICE*Pmi*Jpn1 from *P. mirabilis* (naturally resistant to tetracycline) to *E. coli* were conducted as follows: saturated overnight cultures of *P. mirabilis* strains carrying ICE*Pmi*Jpn1 (Amp^R^, Tet^R^, donors) and of *E. coli* MG1655 Δ*LacZ*::Cm strain (Cm^R^, recipient) were adjusted to an initial OD_600_ of 0.05 in 20 mL of LB and incubated at 37°C until exponential phase (OD_600_ 0.4–0.5). The donor cultures were centrifuged, resuspended in the same volume of 0.9% saline, and divided into 6 mL aliquots for non-irradiated and UV-C irradiated (20 J/m²) conditions. After UV-C irradiation, both aliquots were centrifuged again, resuspended in 6 mL of LB, and mixed with 6 mL culture of the *E. coli* recipients. Each mixture was incubated at 37°C without shaking for 30 minutes, then 10 mL was centrifuged and resuspended in 500 µL of LB. The suspension was inoculated onto 0.22 µm nitrocellulose membranes (MF-Millipore GSWP04700) on LB agar plates and incubated at 37°C. After 24 hours, the membranes were transferred into 5 mL of LB and vigorously shaken until the bacteria suspension was homogeneous. Serial dilutions were performed for plating 100 µL of this material onto LB agar with ampicillin (100 µg/mL) and chloramphenicol (34 µg/mL) to determine the number of transconjugants, 10 µL onto LB agar with tetracycline (12.5 µg/mL) to determine the number of donors, and 10 µL onto LB agar with chloramphenicol (34 µg/mL) to determine the number of recipients. After 24 hours at 37°C, the number of colony-forming units (CFU) of *E. coli* (Amp^R^, Cm^R^, transconjugants), *P. mirabilis* (Tet^R^, donors), and *E. coli* (Cm^R^, recipients) was scored. The conjugation frequency was determined by the ratio of transconjugants/donors.

### Competition assays

Saturated cultures of *P. mirabilis* strains containing ICE*Pmi*Jpn1 and *E. coli* MG1655 Rif^R^ were centrifuged, concentrated in 500 µL of LB medium, and co-inoculated at a 1:1 ratio onto 0.22 µm nitrocellulose membranes (MF-Millipore GSWP04700) placed on LB agar plates. At the initial point and after 3 hours of competition, 10 µL aliquots were collected, serially diluted, and inoculated onto MacConkey agar plates containing tetracycline (12.5 µg/mL) to count *P. mirabilis* CFU and onto plates with rifampicin (24 µg/mL) to determine *E. coli* CFU. The CFU quantities per 1 mL of both microorganisms were evaluated before and after 3 hours of competition.

### Quantification of the relative amount of excised circular ICE DNA

Total DNA was extracted from saturated cultures of *P. mirabilis* strains containing ICE*Pmi*Jpn1 using the Wizard Genomic DNA Purification Kit (Promega, Madison, Wisconsin, USA). For the quantification of the excised circular ICE, qPCRs (quantitative real-time PCR) were performed in the StepOnePlus device (Applied Biosystems, Waltham, Massachusetts, USA), using SYBR Green PCR Master Mix (Applied Biosystems) and oligonucleotides for *rumB* (total ICE control gene): rumBF (5′ GATGGCACCACGAGTA GAGG 3′) and rumBR (5′ TTGTCCGAACTCGACAAGGG 3′) (28); and for the excised circular ICE: SXTJF (5′ GCGAAGGACCTTTGCTATCATC 3′) and SXTJR (5′ TGGTTTTAAGCGTTGAAAGGC 3′) (25), with biological triplicates and three technical replicates for each condition. Each reaction contained 6 µL of SYBR Green, 500 nM of each oligonucleotide, and 5 ng of gDNA, completing the final volume of 12 µL with HyClone Pure Water. The DNA proportion was calculated by the Pfaffl method (43), used to calculate the 2^−∆∆ct^ of primers with differences in efficiency. The formula was: $Ratio={\left(E_{SXTJ}\right)}^{\left(∆CtSXTJ\right)}/{\left(E_{rumB}\right)}^{\left(∆CtrumB\right)}$, where “*E*” refers to primer efficiency and $\Delta Ct={Ct}_{PmBR51-ICE}\;-Ct_{Strains}$.

### RT-qPCR analysis of gene expression

RNA extraction was carried out using TRIzol reagent (Invitrogen, Waltham, Massachusetts, USA) according to the manufacturer's instructions. Extractions were performed on exponential phase cultures (OD_600_ 0.4–0.5) of *P. mirabilis* strains carrying ICE*Pmi*Jpn1, both before and 30 minutes after UV-C irradiation (20 J/m²), to assess variations in basal and induced expression of genes related to ICE conjugation and SOS response. Each RNA sample was treated with RNase-free DNase I (Thermo Fisher Scientific) to remove gDNA contamination. The RNA was then reverse-transcribed into cDNA using the High-Capacity cDNA Reverse Transcription Kit (Thermo Fisher Scientific), following manufacturer's protocol.

Gene expression was quantified by RT-qPCR (reverse transcription quantitative real-time PCR) on a StepOnePlus device (Applied Biosystems), using SYBR Green PCR Master Mix (Applied Biosystems) and specific oligonucleotides for the genes *rpoB* (endogenous control), *recA*, *setC*, and *xis*. The sequences of the oligonucleotides used were: rpoBF (5′ TTAGTTCCTGAGCGTCTGCG 3′), rpoBR (5′ GACGAGCAGTAATACGGCGA 3′), recAF (5′ TAACCCAGAAACCACGACCG 3′), recAR (5′ GAGCCAATGCGACGAATGTC 3′), setCF (5′ GAGCCAATGCGACGAATGTC 3′), setCR (5′ AATGATGAGGTCGCCACTCG 3′), xisF (5′ CTCATACTCCTCCAAGTCTT 3′), and xisR (5′ GGCGAAGAATAAAAGATGGC 3′). Each reaction mixture contained 6 µL of SYBR Green, 500 nM of each oligonucleotide, and 1 µL of cDNA, with UltraPure DEPC-Treated Water (Invitrogen) added to a final volume of 12 µL. Relative gene expression was calculated by the Pfaffl method (43), using the formula: $Relative\;gene\;expression={\left(E_{gene\;of\;interest}\right)}^{\left(\Delta Ct_{gene\;of\;interest}\right)}/{\left(E_{rpoB}\right)}^{\left(\Delta CtrpoB\right)}$, where “*E*” refers to primer efficiency of each oligonucleotide used. For non-irradiated and UV-C irradiated expression levels, we used the $\Delta Ct=Ct_{PmBR51-ICE}\;-Ct_{Strains}$. Whereas relative expression of non-irradiated vs. UV-C irradiated conditions for each strain was calculated using $\Delta Ct=Ct_{non-irradiated}\;-Ct_{UV-C\;irradiated}.$

### Statistical analysis

To perform the statistical analysis, we used the GraphPad Prism software (version 8.0.1) and considered *P* ≤ 0.05 for significant differences. The analysis of mean biofilm production and swarming migration was performed using the unpaired Student's *t* test. The evaluation of *G. mellonella* larvae survival curves after inoculation was performed through Survival Analysis, considering the log-rank test (Mantel-Cox). Analysis of persister formation was made on the counts of 168 and 192 h using unpaired Student's *t* test. Conjugative transfer frequency, circular ICE quantification, and gene expression were evaluated by unpaired Student's *t* test. The correlation of conjugative transfer frequency and the amount of excised ICE DNA were performed using Pearson correlation coefficient.

## RESULTS

We analyzed the effects of ICE*Pmi*Jpn1 on different aspects of *P. mirabilis* physiology, using strains ATCC25933 and PmBR622 with and without the element in pairwise comparisons. For simplicity, their isogenic strains carrying ICE*Pmi*Jpn1 are referred to as ATCC25933-ICE and PmBR622-ICE throughout the text and in figures. Additional analyses with strains PmBR574, PmBR28, and PmBR51 and their derivatives containing ICE*Pmi*Jpn1 were also made in selected experiments.

### Influence of ICE*Pmi*Jpn1 on physiology and pathogenesis of *P. mirabilis*

**Fig 1:**
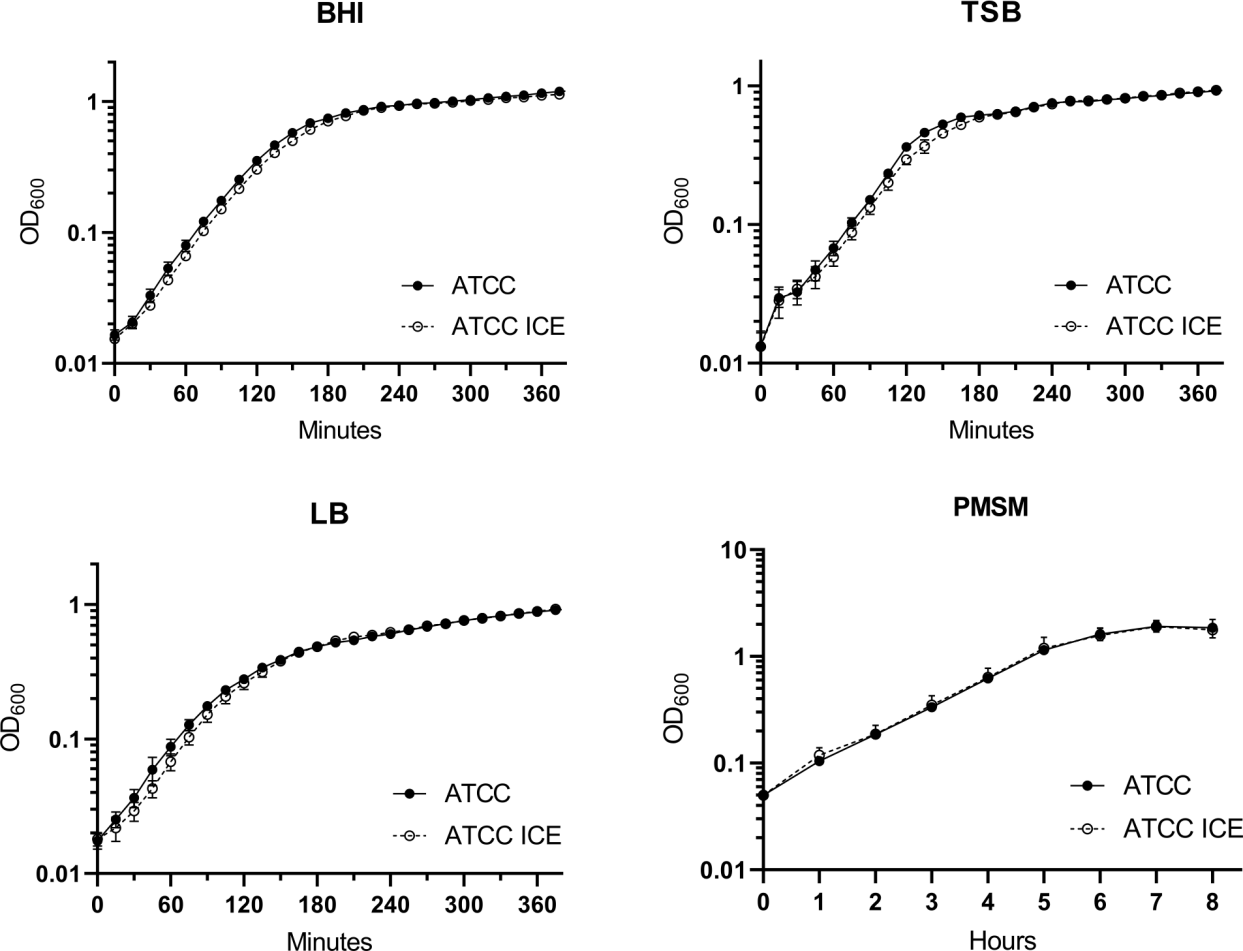
Growth of *Proteus mirabilis* ATCC25933 and ATCC25933-ICE in BHI, TSB, LB, and PMSM media. Growth measured by OD_600_. Mean values of three biological experiments, each with two technical replicates. Error bars represent the standard error of the mean.

**Fig 2:**
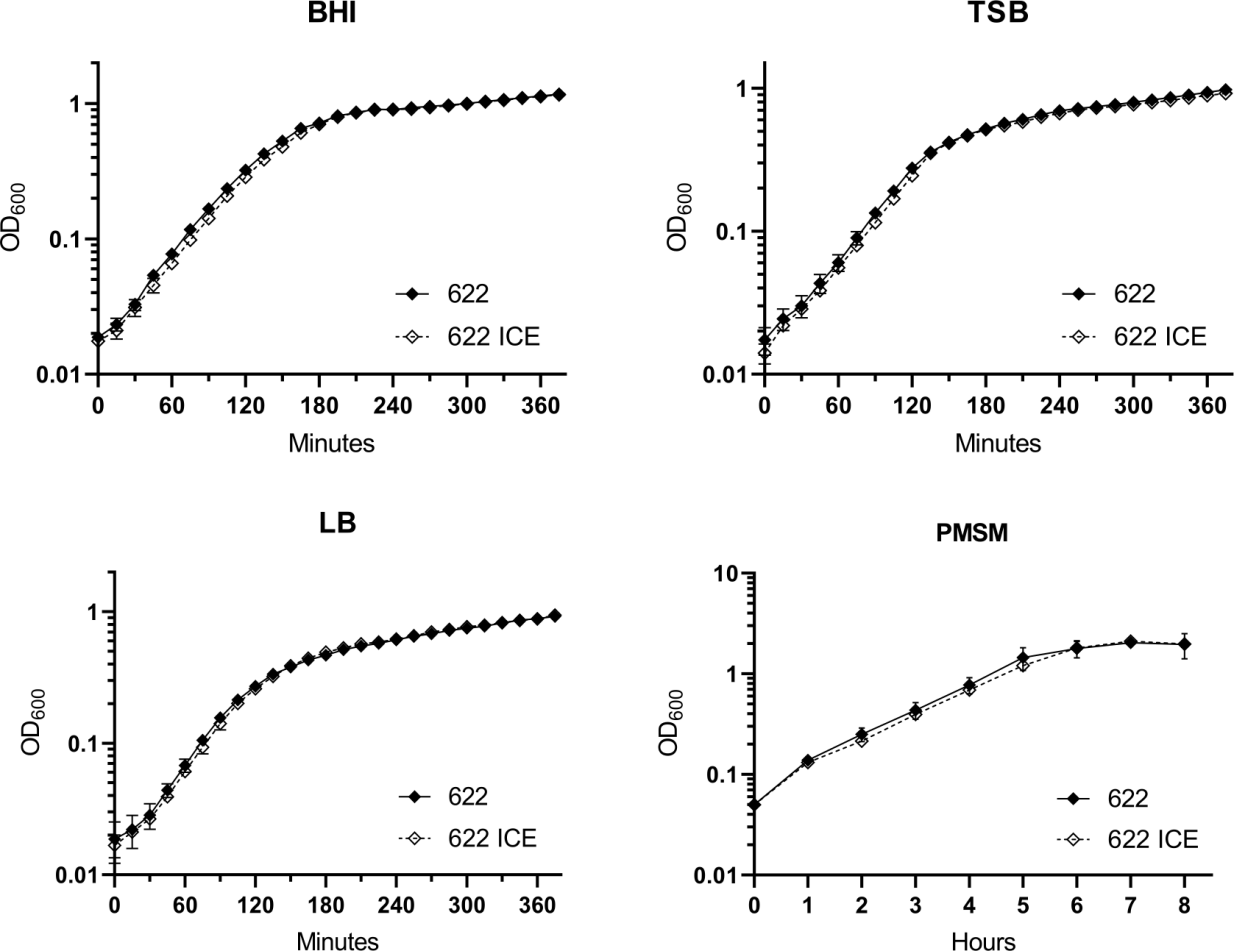
Growth of *Proteus mirabilis* PmBR622 and PmBR622-ICE in BHI, TSB, LB, and PMSM media. Growth measured by OD_600_. Mean values of three biological experiments, each with two technical replicates. Error bars represent the standard error of the mean.

To evaluate whether integration of ICE*Pmi*Jpn1 impacts the fitness of *P. mirabilis*, we analyzed the growth rate of the isogenic strains in various culture media. Growth measurements were conducted in BHI, TSB, LB, and PMSM media to assess any changes linked to the presence of the ICE (Fig. 1 and 2; Fig. S1). The resulting growth curves revealed no differences between the pairs of isogenic strains, in any of the culture media analyzed. These findings suggest that the presence of ICE*Pmi*Jpn1 does not impose a significant fitness cost on *P. mirabilis*. However, while the growth curves show no apparent differences, it is reasonable to assume a minimal metabolic burden associated with the ICE due to the increased use of resources required for its maintenance. This burden, though not visibly reflected in the growth dynamics, may still represent a subtle cost to the cell.

**Fig 3:**
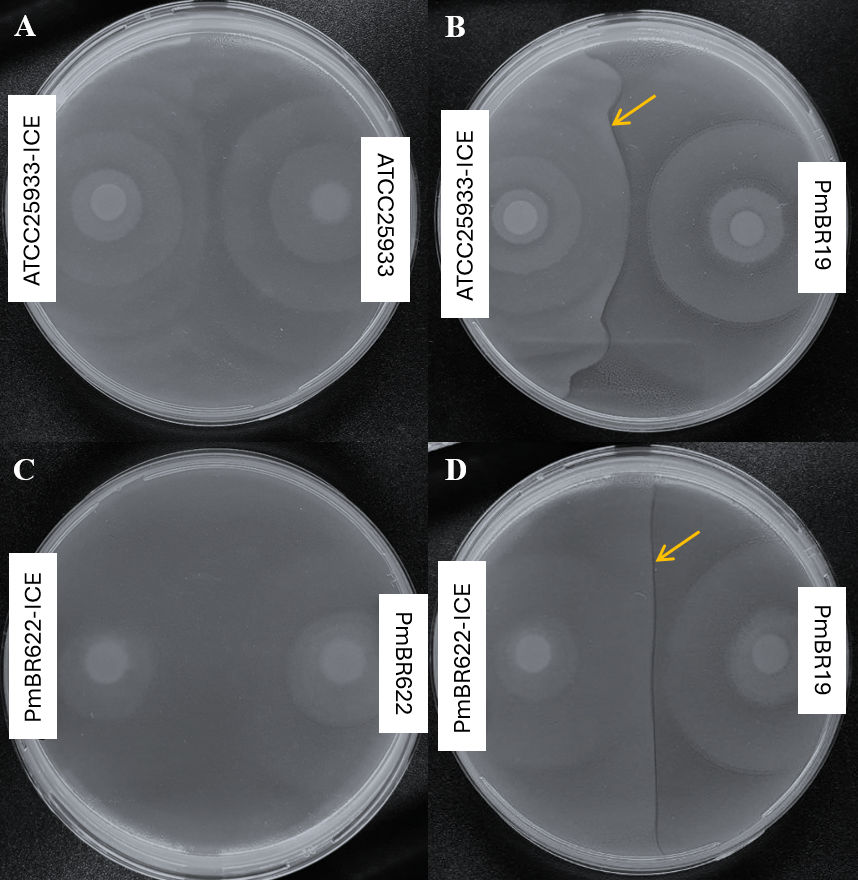
Effect of ICE*Pmi*Jpn1 on self-recognition and formation of Dienes' lines during swarming motility. Co-cultures of *Proteus mirabilis* strains ATCC25933 and PmBR622 carrying ICE*Pmi*Jpn1 with their parental strains (**A, C**) and with PmBR19 strain (**B, D**). Orange arrows indicate the formation of Dienes' lines.

Given the ability of *P. mirabilis* to recognize whether other cells are self or non-self during swarming, we next investigated if the presence of ICE*Pmi*Jpn1 interferes with this phenomenon. To explore this, we inoculated two different strains on the same plate and observed whether competition occurred, resulting in Dienes' line formation, or if the swarms intersected as a sign of self-recognition. Our observations showed that strains carrying ICE*Pmi*Jpn1 consistently formed Dienes' lines when inoculated with PmBR19 (also carrying the same element). Moreover, when these strains were paired with their isogenic counterparts lacking the ICE, self-recognition occurred, leading to swarm merging (Fig. 3; Fig. S2). These results demonstrate that ICE*Pmi*Jpn1 does not interfere with the self-recognition mechanism mediated by the type VI secretion system (T6SS).

**Fig 4:**
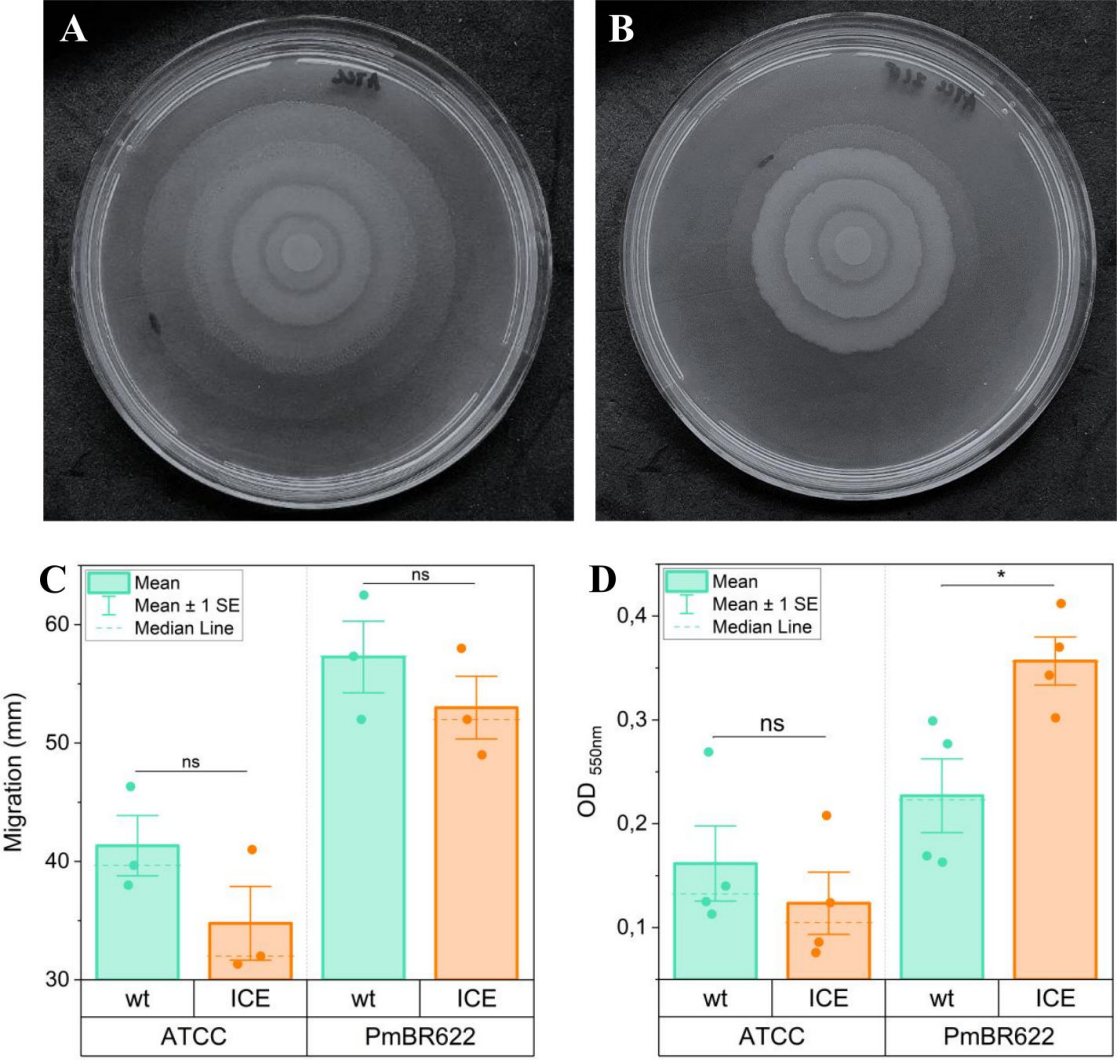
Effect of ICE*Pmi*Jpn1 on swarming motility and biofilm formation of *P. mirabilis*. (A, B) Representative results of swarming motility for a pair of isogenic strains (A: ATCC25933, B: ATCC25933-ICE). (C) Comparison of swarming migration distances (in millimeters) between isogenic strain pairs. Error bars indicate standard error of the mean from three independent experiments, each with three technical replicates. (D) Comparison of biofilm biomass formation, quantified by crystal violet staining measured at OD_550_. Error bars represent the standard error of the mean from four independent experiments, each with eight technical replicates. *P ≤ 0.05, ns: statistically not significant.

To assess whether the acquisition of ICE*Pmi*Jpn1 influences swarming motility, we compared the motility of isogenic strain pairs. After growth in LB medium with 1.5% agar, the distance covered from the point of inoculation to the swarm margin was measured. The average distance of the swarms was compared between isogenic strains, showing that the presence of ICE*Pmi*Jpn1 decreased the swarming motility (Fig. 4A through C), and although there is a trend of slightly less swarming of cells carrying ICE*Pmi*Jpn1, the difference is not statistically significant. Extending this analysis to other strains confirmed that none of them show a reduction in swarming when carrying ICE*Pmi*Jpn1 (Fig. S3A).

We further investigated the potential effect of ICE*Pmi*Jpn1 on biofilm formation by quantifying the biofilm biomass produced by isogenic strain pairs in 96-well plates stained with crystal violet. While no significant difference was observed between ATCC25933 and ATCC25933-ICE strains, PmBR622-ICE produced nearly twice the biofilm mass of its isogenic strain without the ICE, with the difference being statistically significant (*P* = 0.0301) (Fig. 4D). In all other strains tested, the presence of ICE*Pmi*Jpn1 did not alter biofilm formation (Fig. S3B). These results suggest that ICE*Pmi*Jpn1 may enhance biofilm formation depending on the strain genetic background.

**Fig 5:**
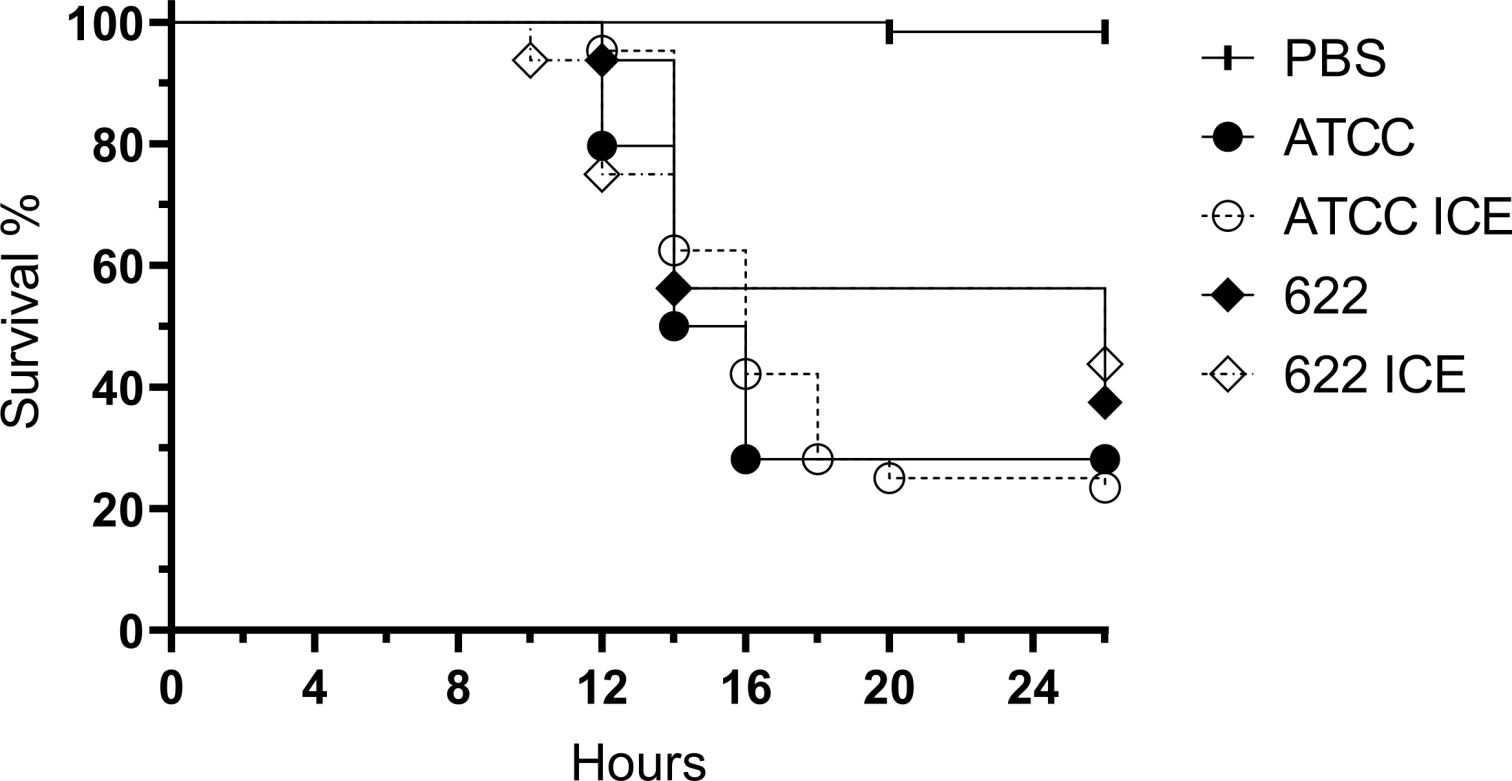
Survival curve of *Galleria mellonella* larvae infected with *Proteus mirabilis* ATCC25933 and PmBR622 with or without ICE*Pmi*Jpn1. Infection was carried out with approximately 10² CFU. Data from four independent experiments for infections with ATCC and ATCC-ICE strains, and one experiment for infection with PmBR622 and PmBR622-ICE strains. *n* = 16 per group in each experiment. PBS: larvae injected with sterile PBS solution.

The influence of ICE*Pmi*Jpn1 on pathogenicity was evaluated by infecting *Galleria mellonella* larvae with isogenic pairs of *P. mirabilis* strains. Rapid mortality of the larvae was observed, beginning within 12 hours post-infection (Fig. 5). However, no significant differences were detected between the isogenic pairs, indicating that the presence of ICE*Pmi*Jpn1 neither increased nor decreased the pathogenic capacity of *P. mirabilis* in the *G. mellonella* model.

**Fig 6:**
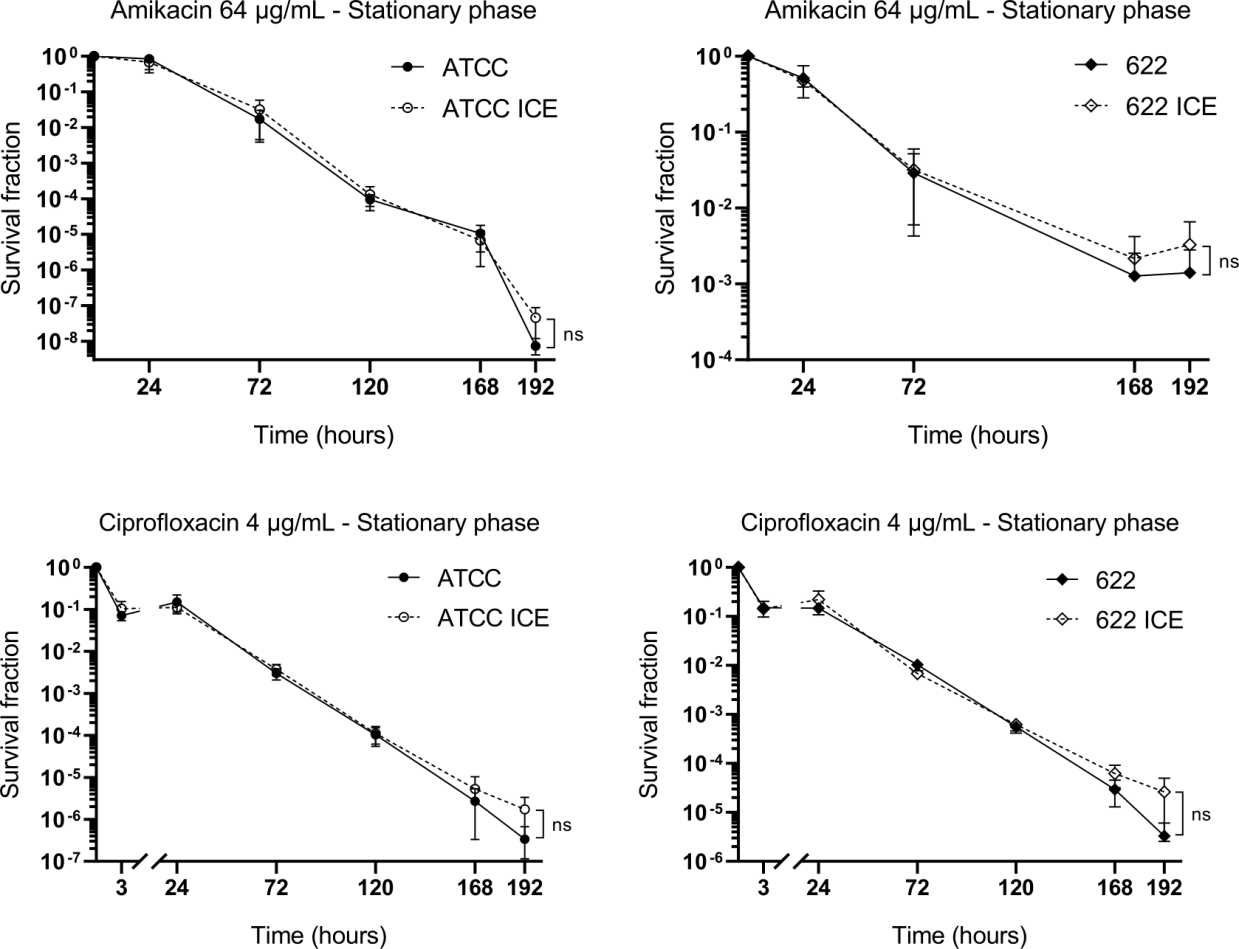
Effect of ICE*Pmi*Jpn1 on antibiotic killing of *P. mirabilis* ATCC25933 and PmBR622 following treatment initiated in the stationary phase. Each dot represents the mean of at least three experiments, with two replicates in each experiment. Bars represent the standard error of the mean. ns: statistically not significant.

**Fig 7:**
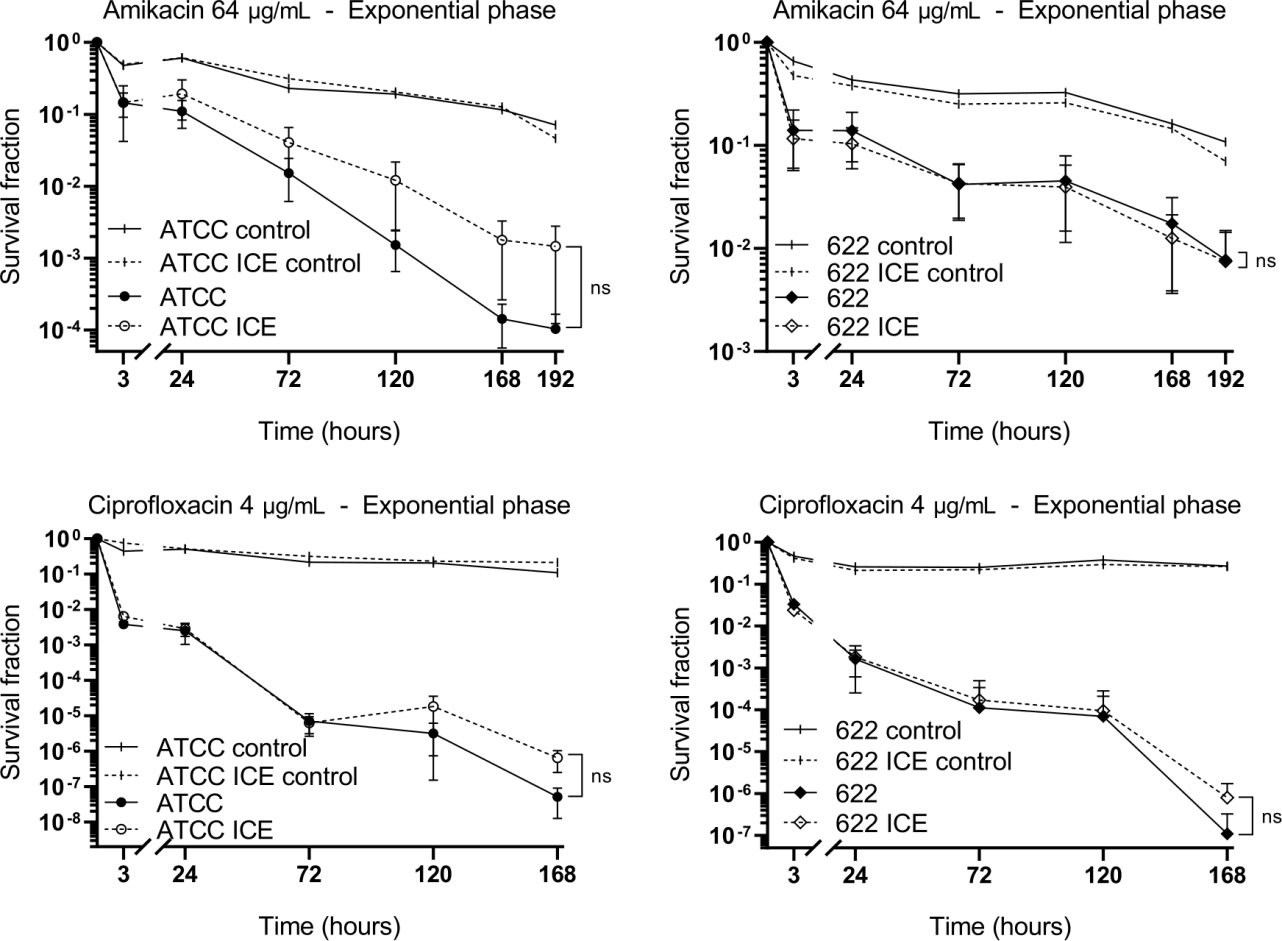
Effect of ICE*Pmi*Jpn1 on antibiotic killing of *P. mirabilis* ATCC25933 and PmBR622 following treatment initiated in the exponential phase. Each dot represents the mean of at least three experiments, with two replicates in each experiment. Bars represent the standard error of the mean. ns: statistically not significant.

Finally, we compared the frequency of persistent cells by monitoring viable cell counts at various time points during treatment with high concentrations of amikacin (64 µg/mL) or ciprofloxacin (4 µg/mL), performed in both stationary and exponential growth phases. All strains tested were susceptible to these antimicrobials: ATCC25933 and ATCC25933-ICE had MIC values of 4 µg/mL and 0.015 µg/mL for amikacin and ciprofloxacin, respectively, while PmBR622 and PmBR622-ICE had MIC values of 8 µg/mL for amikacin and 0.031 µg/mL for ciprofloxacin. The decline in viable cell counts occurred at similar rates regardless of ICE*Pmi*Jpn1 presence as treatment progressed (Fig. 6 and 7). Strains carrying the ICE exhibited slightly higher survival rates at later stages, but the differences were not statistically significant. These findings indicate that the presence of ICE*Pmi*Jpn1 does not substantially impact the frequency of persister cells in *P. mirabilis*, in spite of the presence of TA modules in the element.

### Influence of strain genetic background on ICE*Pmi*Jpn1 transfer frequency

Next, we assessed the efficiency of conjugative transfer of ICE*Pmi*Jpn1 in experiments where different *P. mirabilis* strains harboring ICE*Pmi*Jpn1 were mated with recipient *E. coli* MG1655 cells, under both spontaneous (non-irradiated) and damage-induced conditions (irradiated with 20 J/m² UV-C). The cascade of events leading to conjugative transfer of SXT/R391 elements is triggered under DNA damaging conditions, in which RecA mediates the cleavage of the SetR repressor (27). As a consequence, the *setCD* genes are induced, and their products activate the expression of several genes related to the transmission of the element, such as the ones encoding the excisionase (*xis*) that promotes its excision from the chromosome and the type IV machinery genes (17, 25).

**Fig 8:**
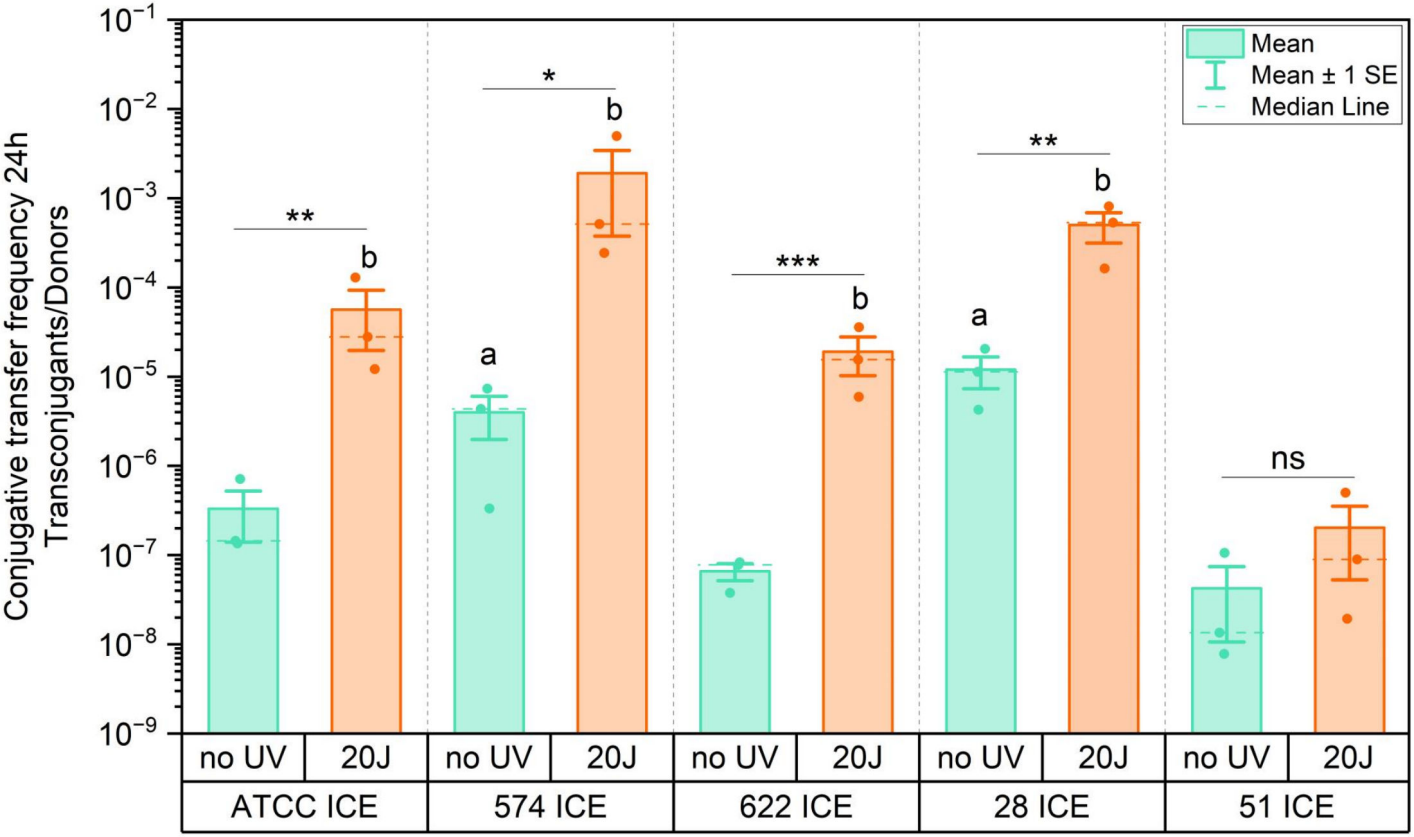
Conjugative transfer frequencies of ICE*Pmi*Jpn1 from different *P. mirabilis* donor strains to *E. coli* recipients in 24 hour mating experiments. Each dot represents the frequency (transconjugants per donor) of an independent experiment, with data including both spontaneous and induced conjugation (following 20 J/m^2^ UV-C irradiation). Bars represent the mean, and error bars represent the standard error. *P ≤ 0.05, **P ≤ 0.01, ***P ≤ 0.001, ns: statistically not significant. (a) statistical difference to spontaneous PmBR51-ICE conjugative transfer frequency. (b) Statistical difference to UV-induced PmBR51-ICE conjugative transfer frequency.

We observed that each strain containing ICE*Pmi*Jpn1 transmits the element at a different frequency, as shown in Fig. 8. Conjugative transmission of ICE*Pmi*Jpn1 varied up to ~280 fold between the different donors in non-irradiated conditions (PmBR28-ICE × PmBR51-ICE), ranging from 4.2 × 10^−8^ to 1.2 × 10^−5^, while UV-C irradiated conjugation exhibited an even greater difference, of >9,000 fold, between PmBR574-ICE and PmBR51-ICE (ranging from 2 × 10^–7^ to 1.9 × 10^–3^). Notably, all strains except PmBR51-ICE exhibited orders of magnitude increases in conjugation following UV irradiation, consistent with previous reports showing UV-induced conjugation of SXT/R391 elements (27, 28). The lack of UV inducibility in PmBR51-ICE suggests a potential defect in the DNA damage-induced activation of conjugative functions.

Since *P. mirabilis* has mechanisms for eliminating competitors, both independent (44) and dependent on the type VI secretion system (5), we hypothesized that differences in conjugative transfer of ICE could be due to killing of *E. coli* receptors, which might reduce the number of recipient cells and thus hinder conjugation. We carried out experiments using two strains, PmBR28-ICE and PmBR622-ICE, that display large differences in spontaneous conjugation (~180 fold), using shorter incubation periods of incubation of the conjugation mixtures (3 h). It is clear that the pronounced difference remains under these conditions (Fig. S4A). Furthermore, we measured the numbers of donors (*P. mirabilis*) and recipients (*E. coli*) at the start and final moments of the conjugation period (Fig. S4B), observing a ~ 10 fold decrease in recipient *E. coli* counts during conjugation with both PmBR28-ICE and PmBR622-ICE, which does not explain the large differences in ICE transfer rates between these two lineages.

**Fig 9:**
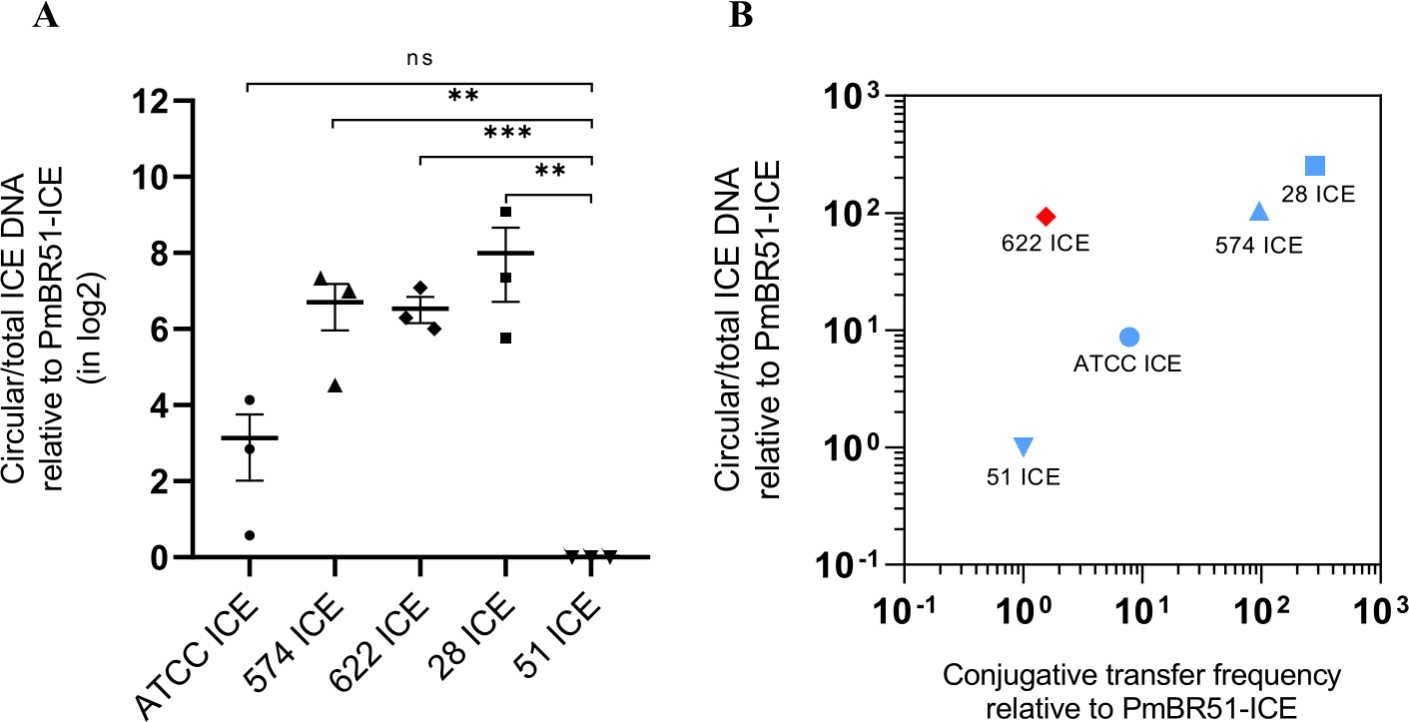
Relative quantification of circular ICE*Pmi*Jpn1 DNA in different *Proteus mirabilis* strains by qPCR. qPCR experiments were performed in three independent biological replicates, each with three technical replicates, each dot represents an experiment, and error bars represent the standard error of the mean. (A) Amount of circular ICE DNA (determined by PCR amplification of the junction) normalized by total ICE DNA present in the cells, relative to strain PmBR51-ICE. (B) Amount of circular ICE DNA normalized by the total ICE DNA present in the cells, compared with conjugative transfer frequencies from Fig. 8. **P ≤ 0.01, ***P ≤ 0.001, ns: statistically not significant.

To confirm these results and understand the molecular mechanisms behind the differences in conjugation between the different *P. mirabilis* strains, we performed experiments to quantify the relative proportion of circular ICE to total ICE in DNA preparations from all strains used in the experiments shown in Fig. 8. Circular ICEs are intermediates in the transfer process, which are formed when the element is excised from the chromosome for subsequent conjugation with recipient cells. We used qPCR to determine the relative amount of circular ICE form. The results, shown in Fig. 9, indicate that most of the strains exhibit a higher proportion of circular ICE DNA compared to PmBR51-ICE (Fig. 9A), a strain characterized as a poor conjugative donor (Fig. 8). Notably, there is a general trend indicating larger amounts of excised ICE in more active conjugative donors, such as PmBR28-ICE (Fig. 9B). However, despite the high correlation coefficient (0.93) observed in the data, the strain PmBR622-ICE deviates from this pattern. It demonstrates a low spontaneous conjugation frequency yet exhibits a higher proportion of excised elements compared to more efficient donors like ATCC25933-ICE.

**Fig 10:**
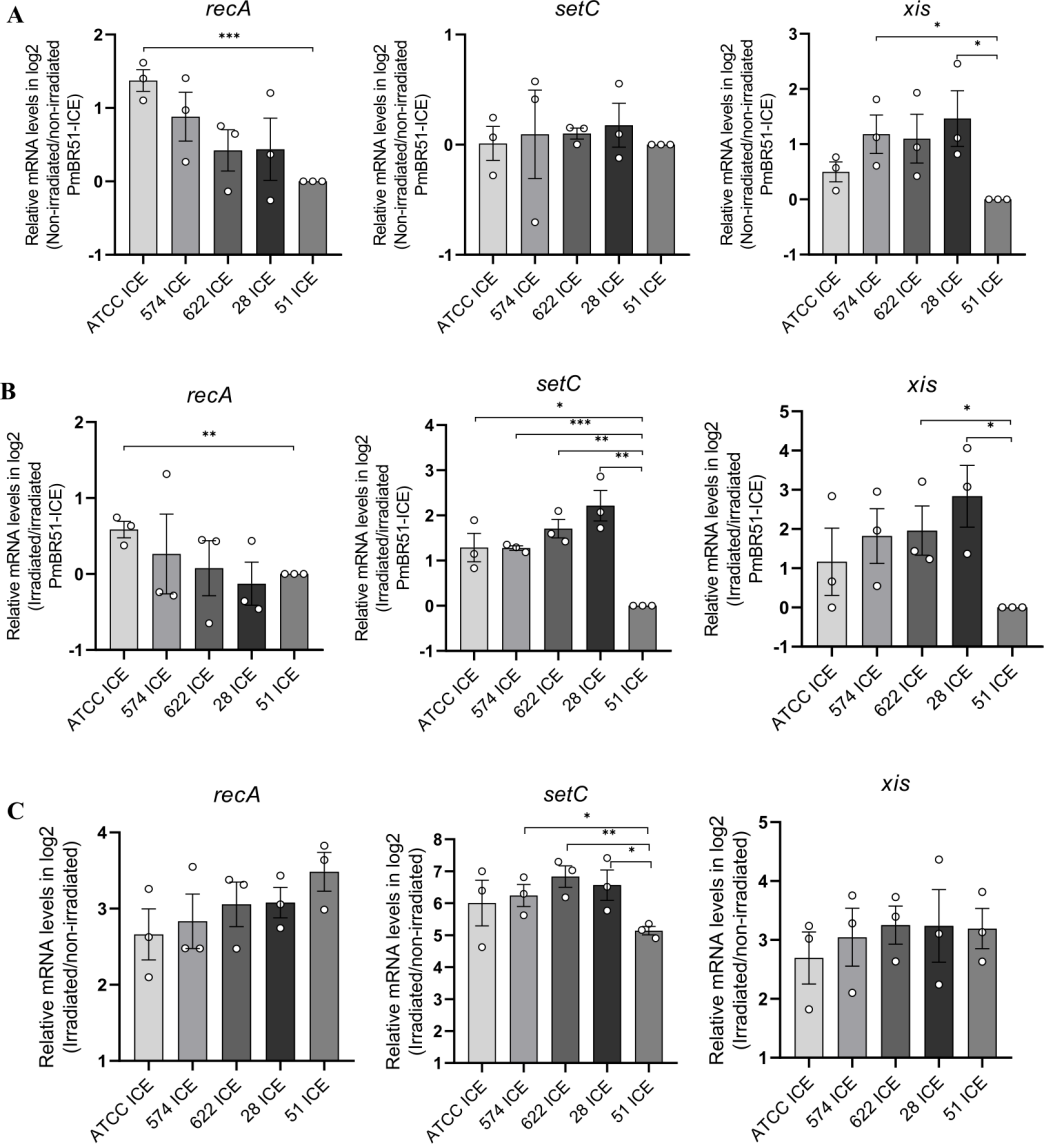
Relative transcript levels of *recA*, *setC,* and *xis* genes measured by RT-qPCR in different *P. mirabilis* strains carrying ICE*PmiJ*pn1. RT-qPCR experiments were performed using three independent biological replicates, each with two technical replicates. Each dot represents an individual experiment, bars represent the mean, and error bars represent the standard error of the mean. Transcript levels were normalized by the housekeeping gene *rpoB*. (A) Non-irradiated expression levels relative to PmBR51-ICE. (B) Irradiated (20 J/m² UV-C) expression levels relative to PmBR51-ICE. (C) Relative expression levels of UV-C irradiated strains compared to their non-irradiated controls. *P ≤ 0.05, **P ≤ 0.01, ***P ≤ 0.001.

To determine if the large differences in conjugation capacities are associated with differences in the expression of regulatory genes, we compared the expression levels of *recA*, *setC*, and *xis* across the different lineages, relative to PmBR51-ICE, which exhibits the lowest conjugation capacity (Fig. 10A and B). *recA* is a chromosomal gene part of the SOS response of enterobacteria, while *setC* and *xis* are ICE genes upregulated in response to DNA damage (27, 29, 30).

In terms of basal expression levels of the three genes (Fig. 10A), there are minor differences detected when different strains are compared. There is no statistically significant variation of *setC* expression among all strains; only strain ATCC25933-ICE shows a modestly increased expression of *recA*, and strains PmBR574-ICE and PmBR28-ICE show a significant increase in *xis* levels. The last observation is in agreement with the fact that PmBR574-ICE and PmBR28-ICE are the ones with statistically significant increases in spontaneous conjugative frequency when compared to PmBR51-ICE, the least effective donor (Fig. 8). On the other hand, analyses of the levels of *xis* and specially *setC* after DNA damage induced by UV-C irradiation (Fig. 10B) show that all strains have higher expression than PmBR51-ICE, which may explain why this particular strain is unable to induce ICE conjugation after DNA damage. In Fig. 10C, it can be observed that PmBR51-ICE has a normal induction of the SOS response, as indicated by similar levels of *recA* induction by UV-C, but a lower induction of the master regulator *setC* than most strains.

Altogether, these results indicate that all strains activate the SOS response similarly, but PmBR51-ICE induces *setCD* to a lesser extent than the other strains, which may contribute to its lack of damage-inducible conjugation (Fig. 8). Nevertheless, the differences in conjugation between the other *P. mirabilis* strains as donors are not fully explained by significant differences in the expression of the genes analyzed, although higher basal levels of *xis* do correlate with higher conjugative transfer capacity of strains PmBR574-ICE and PmBR28-ICE.

## DISCUSSION

Despite significant advances in understanding MGEs, studies on ICEs remain relatively recent, as these elements were only reclassified in the early 2000s (19). Research has primarily focused on their distribution, host range, resistance genes, and regulatory mechanisms (17, 26, 28, 45–47), but their biological impact on natural hosts remains underexplored (12).

MGEs often impose a fitness cost on their host due to the metabolic burden of replication and gene expression. In the absence of selective pressure, such elements can be lost (48). However, our findings indicate that ICE*Pmi*Jpn1 does not affect *P. mirabilis* fitness across different media, including minimal media, suggesting minimal metabolic impact. This likely explains its stable maintenance within *P. mirabilis* populations despite the absence of obvious adaptive traits.

A defining feature of *P. mirabilis* is its ability to swarm, a specialized motility mechanism involving differentiation into elongated, hyperflagellated cells for coordinated migration (1, 2, 8). Swarming is influenced by environmental triggers and requires precise regulation of genes related to cell morphology, metabolism, and respiration (49–51). This behavior provides a competitive advantage in polymicrobial environments and contributes to pathogenicity by facilitating surface colonization (5, 49, 50, 52).

Our results showed that strains carrying ICE*Pmi*Jpn1 were not significantly impaired in swarming motility based on the average of multiple experiments. However, a slight decrease was observed in each individual experiment (see representative results in Fig. 4A and B), which may be attributed to pleiotropic effects associated with the ICE. One possible explanation is that the large size of ICE*Pm*iJpn1 could interfere with transcriptional regulation, subtly affecting gene expression during swarming. Additionally, ICE-encoded pilins and pili regulators may influence cell surface properties, potentially altering swarming dynamics (53, 54). While these effects were not statistically significant, they suggest a potential link between ICE carriage and swarming regulation that warrants further investigations.

During swarming, *P. mirabilis* expresses T6SS-mediated defense mechanisms, inserting lethal toxins into competing strains or species, forming Dienes' lines with non-self strains and enabling self-recognition (5). In our experiments, strains carrying ICE*Pmi*Jpn1 maintained their ability to recognize isogenic strains and form Dienes' lines with PmBR19, indicating that T6SS activity remained unaffected by the ICE's presence.

Swarming and biofilm formation are inversely related processes in *P. mirabilis*. While swarming involves flagellar synthesis and cell elongation, biofilm formation relies on fimbriae and adhesins for surface attachment (55, 56). Most ICE*Pmi*Jpn1-carrying strains exhibited no changes in biofilm formation, except for PmBR622-ICE, which showed a significant increase in biofilm biomass. This suggests that ICE*Pmi*Jpn1 may confer a strain-dependent advantage for biofilm formation, potentially facilitating ICE dissemination in specific hosts.

To assess whether ICE*Pmi*Jpn1 affects *P. mirabilis* pathogenicity, we used *Galleria mellonella* larvae as an invertebrate infection model. Survival curves showed that ICE*Pmi*Jpn1 did not alter the pathogenicity of *P. mirabilis*, as both ICE-carrying and ICE-lacking strains exhibited similar pathogenic potential. These results align with previous studies showing that *P. mirabilis* infections cause rapid larval mortality (57), faster than pathogens such as *Staphylococcus aureus*, which takes several days to effectively eliminate all larvae (58). While our data indicate that ICE*Pmi*Jpn1 does not impact the pathogenicity of *P. mirabilis*, further studies using other infection models should be tested.

Since ICE*Pmi*Jpn1 harbors *hipAB*, a toxin/antitoxin module associated with bacterial persistence (59), we investigated whether the ICE influenced persister cell formation. Persisters may be categorized into two cell types: type I, which enter dormancy in response to external stresses as during stationary phase, and type II, which stochastically enter dormancy during exponential growth (60). However, no significant differences were observed between isogenic pairs in either stationary-phase or exponential-phase persister assays. This suggests that *hipAB* primarily contributes to ICE stability, as shown previously for ICER391 (61), rather than modulating persistence, in the context of ICE*Pmi*Jpn1 within *P. mirabilis*.

ICE*Pmi*Jpn1 conjugative transfer varied significantly among *P. mirabilis* strains, with transfer efficiency differing by up to ~280 fold. This variation persisted under DNA-damaging conditions known to stimulate element transmission (27, 28) and was not linked to interspecies competition with *E. coli*. Instead, transfer efficiency correlated with circularized ICE levels, except for PmBR622-ICE, which exhibited high circular ICE levels but low transfer rates.

Multiple factors may explain differences in ICE circularization and transfer, including variations in the persistence of the element as an extrachromosomal autonomous replicating unit (61), expression of toxin-antitoxin (TA) modules (62) and regulatory genes, the presence of additional mobilizable elements (including other ICEs) in the host (63), and lineage-specific differences in SOS response activation, since a more active SOS response could enhance ICE transfer and influence ICE circularization across lineages. While most strains exhibited similar *recA* expression, *xis* expression correlated with spontaneous conjugation efficiency—strains with higher *xis* expression (PmBR574-ICE and PmBR28-ICE) exhibited increased transfer, whereas PmBR51-ICE displayed the lowest *xis* expression and conjugation frequency. Additionally, reduced *setC* expression in PmBR51-ICE following UV-C exposure suggests a diminished ability to activate conjugation under DNA-damaging conditions. SetCD are the positive regulators of genes involved in conjugation (17), and reduced *setC* expression may contribute to the strain's lower UV-induced conjugation. The underlying cause of this diminished *setC* expression remains unclear, given that the SOS response is active in PmBR51-ICE (*recA* expressed), and this strain exhibits high UV-mutagenic capacity when carrying ICE*Pmi*Jpn1 (28).

Interestingly, the conjugative profile of PmBR622-ICE presents an exception. Despite having a high proportion of circularized ICE and comparable expression of conjugation-related genes to more efficient donor strains like PmBR574-ICE and PmBR28-ICE, it remains a poor donor of the ICE. For most other strains, however, conjugation efficiency correlates with the proportion of circularized ICEs, with the most efficient donors exhibiting higher levels of circularized elements. This suggests that, beyond circularization, additional host-specific regulatory factors or structural constraints may influence conjugation efficiency. The observed variability across strains highlights the multifactorial nature of ICE maintenance and transfer, highlighting the importance of host-ICE interactions in determining transmission dynamics.

Previous studies have shown that elements like R391 can persist in a plasmid-like state (61). However, since our study examines the same element (ICE*Pmi*Jpn1) across different strains, our findings suggest that host strain-specific factors, rather than only ICE-intrinsic or species-specific mechanisms, influence ICE circularization, conjugation, and maintenance within the host chromosome. Overall, the variability in conjugative transfer between strains appears to be a multifactorial phenomenon, warranting further investigation to fully understand the interplay of regulatory, structural, and host-specific factors.

### Conclusions

This study provides valuable insights into the relationship and co-evolution of ICE*Pmi*Jpn1 with its natural host, *Proteus mirabilis*. Our findings demonstrate that ICE*Pmi*Jpn1 imposes minimal to no fitness burden on its host, in contrast to many other mobile genetic elements that often lead to metabolic costs. While a slight reduction in swarming motility was observed in some strains, the overall physiological and pathogenic traits of *P. mirabilis* remained largely unaffected by the presence of ICE*Pmi*Jpn1.

The ability of ICE*Pmi*Jpn1 to enhance biofilm formation in one strain suggests that it may confer context-dependent advantages, potentially facilitating its spread in specific environments. Previous studies have shown that ICEs can confer adaptive functions to *P. mirabilis*, such as increased SOS mutagenesis via functional *rumAB* operon in SXT/R391 ICEs (28) leading to resistance to antimicrobials (64). This supports the notion of a beneficial relationship between ICE*Pmi*Jpn1 and its host, potentially contributing to its widespread distribution in *P. mirabilis* populations.

Our results also highlight the complexity of ICE-host interactions, particularly in the context of conjugative transfer. We observed significant variability in the efficiency of ICE*Pmi*Jpn1 transfer among different *P. mirabilis* strains, even under DNA-damaging conditions that typically induce conjugation. This variability appears to be influenced by host-specific factors that impact the expression of excisionase (*xis*) and the induction of the master regulator *setCD*, rather than only intrinsic properties of the ICE itself.

Understanding additional regulatory mechanisms governing ICE excision, circularization, and conjugation is crucial for predicting and controlling the spread of mobile genetic elements. Future research should focus on the interplay between ICEs and their hosts. Addressing these knowledge gaps will be essential in developing strategies to mitigate the dissemination of multidrug-resistant pathogens.

## Data Availability

All data generated or analyzed during this study are included in this published article and in File S1.

## AFTER EPUB

*[This article was published on 23 May 2025 with an error in the last author's name. The byline was corrected in the current version, posted on 10 June 2025.]*

## SUPPLEMENTAL MATERIAL

Data S1 spectrum.00339-25-s0001.xlsx Raw data.

Fig. S1 spectrum.00339-25-s0002.pdf Growth of *P. mirabilis* in different media.

Fig. S2 spectrum.00339-25-s0003.pdf Self-recognition of different strains during swarming motility.

Fig. S3 spectrum.00339-25-s0004.pdf Swarming motility and biofilm formation of different *P. mirabilis* strains.

Fig. S4 spectrum.00339-25-s0005.pdf Conjugative transfer frequencies of ICE*Pmi*Jpn1 from different *P. mirabilis* donor strains.

ASM does not own the copyrights to Supplemental Material that may be linked to, or accessed through, an article. The authors have granted ASM a non-exclusive, world-wide license to publish the Supplemental Material files. Please contact the corresponding author directly for reuse.

## References

[1] Armbruster CE, Mobley HLT, Pearson MM. 2018. Pathogenesis of Proteus mirabilis infection. EcoSal Plus 8:1–73. 10.1128/ecosalplus.esp-0009-2017 PMC5880328 29424333

[2] Drzewiecka D. 2016. Significance and roles of Proteus spp. bacteria in natural environments. Microb Ecol 72:741–758. doi:10.1007/s00248-015-0720-6 26748500 PMC5080321

[3] Adeolu M, Alnajar S, Naushad S, S Gupta R. 2016. Genome-based phylogeny and taxonomy of the “Enterobacteriales”: proposal for Enterobacterales ord. nov. divided into the families Enterobacteriaceae, Erwiniaceae fam. nov., Pectobacteriaceae fam. nov., Yersiniaceae fam. nov., Hafniaceae fam. nov., Morganellaceae fam. nov., and Budviciaceae fam. nov. Int J Syst Evol Microbiol 66:5575–5599. doi:10.1099/ijsem.0.001485 27620848

[4] Pearson MM, Sebaihia M, Churcher C, Quail MA, Seshasayee AS, Luscombe NM, Abdellah Z, Arrosmith C, Atkin B, Chillingworth T, Hauser H, Jagels K, Moule S, Mungall K, Norbertczak H, Rabbinowitsch E, Walker D, Whithead S, Thomson NR, Rather PN, Parkhill J, Mobley HLT. 2008. Complete genome sequence of uropathogenic Proteus mirabilis, a master of both adherence and motility. J Bacteriol 190:4027–4037. doi:10.1128/JB.01981-07 18375554 PMC2395036

[5] Alteri CJ, Himpsl SD, Pickens SR, Lindner JR, Zora JS, Miller JE, Arno PD, Straight SW, Mobley HLT. 2013. Multicellular bacteria deploy the type VI secretion system to preemptively strike neighboring cells. PLoS Pathog 9:e1003608. doi:10.1371/journal.ppat.1003608 24039579 PMC3764213

[6] Alteri CJ, Mobley HLT. 2016. The versatile type VI secretion system. Virulence Mech Bact Pathog:337–356. doi:10.1128/9781555819286.ch12 PMC4887148 27227310

[7] Harada S, Ishii Y, Saga T, Tateda K, Yamaguchi K. 2010. Chromosomally encoded bla_CMY-2_ located on a novel SXT/R391-related integrating conjugative element in a Proteus mirabilis clinical isolate. Antimicrob Agents Chemother 54:3545–3550. doi:10.1128/AAC.00111-10 20566768 PMC2934980

[8] Schaffer JN, Pearson MM. 2015. Proteus mirabilis and urinary tract infections. Microbiol Spectr 3:1–39. doi:10.1128/microbiolspec.UTI-0017-2013 PMC4638163 26542036

[9] Van den Bergh B, Fauvart M, Michiels J. 2017. Formation, physiology, ecology, evolution and clinical importance of bacterial persisters. FEMS Microbiol Rev 41:219–251. doi:10.1093/femsre/fux001 28333307

[10] Wilmaerts D, Windels EM, Verstraeten N, Michiels J. 2019. General mechanisms leading to persister formation and awakening. Trends Genet 35:401–411. doi:10.1016/j.tig.2019.03.007 31036343

[11] Liu S, Wu N, Zhang S, Yuan Y, Zhang W, Zhang Y. 2017. Variable persister gene interactions with (p)ppGpp for persister formation in Escherichia coli. Front Microbiol 8:1–14. doi:10.3389/fmicb.2017.01795 28979246 PMC5611423

[12] Botelho J, Schulenburg H. 2021. The role of integrative and conjugative elements in antibiotic resistance evolution. Trends Microbiol 29:8–18. doi:10.1016/j.tim.2020.05.011 32536522

[13] Shelenkov A, Petrova L, Fomina V, Zamyatin M, Mikhaylova Y, Akimkin V. 2020. Multidrug-resistant Proteus mirabilis strain with cointegrate plasmid. Microorganisms 8:1–13. doi:10.3390/microorganisms8111775 PMC7696407 33198099

[14] Mata C, Navarro F, Miró E, Walsh TR, Mirelis B, Toleman M. 2011. Prevalence of SXT/R391-like integrative and conjugative elements carrying bla_CMY-2_ in Proteus mirabilis. J Antimicrob Chemother 66:2266–2270. doi:10.1093/jac/dkr286 21752830

[15] Meini S, Tascini C, Cei M, Sozio E, Rossolini GM. 2019. AmpC β-lactamase-producing Enterobacterales: what a clinician should know. Infection 47:363–375. doi:10.1007/s15010-019-01291-9 30840201

[16] Burrus V, Marrero J, Waldor MK. 2006. The current ICE age: biology and evolution of SXT-related integrating conjugative elements. Plasmid 55:173–183. doi:10.1016/j.plasmid.2006.01.001 16530834

[17] Poulin-Laprade D, Matteau D, Jacques P-É, Rodrigue S, Burrus V. 2015. Transfer activation of SXT/R391 integrative and conjugative elements: unraveling the SetCD regulon. Nucleic Acids Res 43:2045–2056. doi:10.1093/nar/gkv071 25662215 PMC4344509

[18] Ryan MP, Carraro N, Slattery S, Pembroke JT. 2024. Integrative Conjugative Elements (ICEs) of the SXT/R391 family drive adaptation and evolution in γ-Proteobacteria. Crit Rev Microbiol 50:105–126. doi:10.1080/1040841X.2022.2161870 36634159

[19] Burrus V, Pavlovic G, Decaris B, Guédon G. 2002. Conjugative transposons: the tip of the iceberg. Mol Microbiol 46:601–610. doi:10.1046/j.1365-2958.2002.03191.x 12410819

[20] Carraro N, Burrus V. 2014. Biology of three ICE families: SXT/R391, ICEBs1, and ICESt1/ICESt3. Microbiol Spectr 2:1–20. doi:10.1128/microbiolspec.MDNA3-0008-2014 26104437

[21] Wozniak RAF, Fouts DE, Spagnoletti M, Colombo MM, Ceccarelli D, Garriss G, Déry C, Burrus V, Waldor MK. 2009. Comparative ICE genomics: insights into the evolution of the SXT/R391 family of ICEs. PLoS Genet 5:e1000786. doi:10.1371/journal.pgen.1000786 20041216 PMC2791158

[22] Johnson CM, Grossman AD. 2015. Integrative and Conjugative Elements (ICEs): what they do and how they work. Annu Rev Genet 49:577–601. doi:10.1146/annurev-genet-112414-055018 26473380 PMC5180612

[23] Sato JL, Fonseca MRB, Cerdeira LT, Tognim MCB, Sincero TCM, Noronha do Amaral MC, Lincopan N, Galhardo RS. 2020. Genomic analysis of SXT/R391 integrative conjugative elements from Proteus mirabilis isolated in Brazil. Front Microbiol 11:571472. doi:10.3389/fmicb.2020.571472 33193168 PMC7606855

[24] Hochhut B, Waldor MK. 1999. Site-specific integration of the conjugal Vibrio cholerae SXT element into prfC. Mol Microbiol 32:99–110. doi:10.1046/j.1365-2958.1999.01330.x 10216863

[25] Burrus V, Waldor MK. 2003. Control of SXT integration and excision. J Bacteriol 185:5045–5054. doi:10.1128/JB.185.17.5045-5054.2003 12923077 PMC181012

[26] Poulin-Laprade D, Burrus V. 2015. A γ Cro-like repressor is essential for the induction of conjugative transfer of SXT/R391 elements in response to DNA damage. J Bacteriol 197:3822–3833. doi:10.1128/JB.00638-15 26438816 PMC4652052

[27] Beaber JW, Hochhut B, Waldor MK. 2004. SOS response promotes horizontal dissemination of antibiotic resistance genes. Nature 427:72–74. doi:10.1038/nature02241 14688795

[28] Sato JL, Fonseca DLH, Galhardo RS. 2022. rumAB genes from SXT/R391 ICEs confer UV-induced mutability to Proteus mirabilis hosts and improve conjugation after UV irradiation. DNA Repair (Amst) 112:103297. doi:10.1016/j.dnarep.2022.103297 35202966

[29] Friedberg EC, Walker GC, Siede W, Wood RD, Schultz RA, Ellenberger T. 2006. The SOS responses of prokaryotes to DNA damage, p 463–508. In DNA repair and mutagenesis. ASM Press, Washington, DC, USA.

[30] Lima-Noronha MA, Fonseca DLH, Oliveira RS, Freitas RR, Park JH, Galhardo RS. 2022. Sending out an SOS - the bacterial DNA damage response. Genet Mol Biol 45:e20220107. doi:10.1590/1678-4685-GMB-2022-0107 36288458 PMC9578287

[31] He J, Sun L, Zhang L, Leptihn S, Yu Y, Hua X. 2021. A novel SXT/R391 integrative and conjugative element carries two copies of the blaNDM-1 gene in Proteus mirabilis. mSphere 6:1–12. doi:10.1128/mSphere.00588-21 PMC8386438 34378988

[32] Han Y, Gao Y-F, Xu H, Li J-P, Li C, Song C-L, Lei C-W, Chen X, Wang Q, Ma B-H, Wang H-N. 2024. Characterization and risk assessment of novel SXT/R391 integrative and conjugative elements with multidrug resistance in Proteus mirabilis isolated from China, 2018–2020. Microbiol Spectr 12:1–20. 10.1128/spectrum.01209-23 PMC10871549 38197656

[33] Ryan MP, Armshaw P, Pembroke JT. 2016. SXT/R391 integrative and conjugative elements (ICEs) encode a novel “trap-door” strategy for mobile element escape. Front Microbiol 7:829. doi:10.3389/fmicb.2016.00829 27303400 PMC4885824

[34] Aberkane S, Compain F, Decré D, Dupont C, Laurens C, Vittecoq M, Pantel A, Solassol J, Carrière C, Renaud F, Brieu N, Lavigne JP, Bouzinbi N, Ouédraogo AS, Jean-Pierre H, Godreuil S. 2016. High prevalence of SXT/R391-related integrative and conjugative elements carrying bla_CMY-2_ in Proteus mirabilis isolates from gulls in the South of France. Antimicrob Agents Chemother 60:1148–1152. doi:10.1128/AAC.01654-15 26643344 PMC4750665

[35] Lei CW, Zhang AY, Wang HN, Liu BH, Yang LQ, Yang YQ. 2016. Characterization of SXT/R391 integrative and conjugative elements in Proteus mirabilis isolates from food-producing animals in China. Antimicrob Agents Chemother 60:1935–1938. doi:10.1128/AAC.02852-15 26824957 PMC4775944

[36] Bie L, Wu H, Wang XH, Wang M, Xu H. 2017. Identification and characterization of new members of the SXT/R391 family of integrative and conjugative elements (ICEs) in Proteus mirabilis. Int J Antimicrob Agents 50:242–246. doi:10.1016/j.ijantimicag.2017.01.045 28602701

[37] Mac Aogáin M, Rogers TR, Crowley B. 2016. Identification of emergent bla_CMY-2_-carrying Proteus mirabilis lineages by whole-genome sequencing. New Microbes New Infect 9:58–62. doi:10.1016/j.nmni.2015.11.012 26865983 PMC4710684

[38] Fonseca MRB, Sato JL, Lima-Noronha MA, Migliorini LB, Fernández-Silva FS, Galhardo RS. 2018. Increased mutability to fosfomycin resistance in Proteus mirabilis clinical isolates. Infect Genet Evol 58:27–33. doi:10.1016/j.meegid.2017.12.012 29248795

[39] Pearson MM. 2019. Proteus mirabilis. Springer, New York, New York, NY.

[40] O’Toole GA. 2011. Microtiter dish biofilm formation assay. J Vis Exp 47:1–2. doi:10.3791/2437 PMC3182663 21307833

[41] Ramarao N, Nielsen-Leroux C, Lereclus D. 2012. The insect Galleria mellonella as a powerful infection model to investigate bacterial pathogenesis. J Vis Exp 70:e4392. doi:10.3791/4392 PMC3567165 23271509

[42] Wiegand I, Hilpert K, Hancock REW. 2008. Agar and broth dilution methods to determine the minimal inhibitory concentration (MIC) of antimicrobial substances. Nat Protoc 3:163–175. doi:10.1038/nprot.2007.521 18274517

[43] Pfaffl MW. 2001. A new mathematical model for relative quantification in real-time RT-PCR. Nucleic Acids Res 29:45. doi:10.1093/nar/29.9.e45 PMC55695 11328886

[44] Kiani D, Santus W, Kiernan KA, Behnsen J. 2021. Proteus mirabilis employs a contact-dependent killing system against competing Enterobacteriaceae. mSphere 6:e0032121. doi:10.1128/mSphere.00321-21 34319125 PMC8386478

[45] Poulin-Laprade D, Carraro N, Burrus V. 2015. The extended regulatory networks of SXT/R391 integrative and conjugative elements and IncA/C conjugative plasmids. Front Microbiol 6:837. doi:10.3389/fmicb.2015.00837 26347724 PMC4542580

[46] Gonzalez M, Huston D, McLenigan MP, McDonald JP, Garcia AM, Borden KS, Woodgate R. 2019. SetRICE391, a negative transcriptional regulator of the integrating conjugative element 391 mutagenic response. DNA Repair (Amst) 73:99–109. doi:10.1016/j.dnarep.2018.11.007 30581075 PMC6737337

[47] McDonald JP, Quiros DR, Vaisman A, Mendez AR, Reyelt J, Schmidt M, Gonzalez M, Woodgate R. 2021. CroSR391, an ortholog of the λ Cro repressor, plays a major role in suppressing polVR391-dependent mutagenesis. Mol Microbiol 116:877–889. 34184328 10.1111/mmi.14777 PMC8460599

[48] San Millan A, MacLean RC. 2017. Fitness costs of plasmids: a limit to plasmid transmission. Microbiol Spectr 5:65–79. doi:10.1128/microbiolspec.mtbp-0016-2017 PMC11687550 28944751

[49] Pearson MM, Rasko DA, Smith SN, Mobley HLT. 2010. Transcriptome of swarming Proteus mirabilis. Infect Immun 78:2834–2845. doi:10.1128/IAI.01222-09 20368347 PMC2876570

[50] Tuson HH, Copeland MF, Carey S, Sacotte R, Weibel DB. 2013. Flagellum density regulates Proteus mirabilis swarmer cell motility in viscous environments. J Bacteriol 195:368–377. doi:10.1128/JB.01537-12 23144253 PMC3553826

[51] Howery KE, Clemmer KM, Rather PN. 2016. The Rcs regulon in Proteus mirabilis: implications for motility, biofilm formation, and virulence. Curr Genet 62:775–789. doi:10.1007/s00294-016-0579-1 26936153

[52] Little K, Austerman J, Zheng J, Gibbs KA. 2019. Cell shape and population migration are distinct steps of Proteus mirabilis swarming that are decoupled on high-percentage agar. J Bacteriol 201:1–15. doi:10.1128/JB.00726-18 PMC6509654 30858303

[53] Kuchma SL, Griffin EF, O’Toole GA. 2012. Minor pilins of the type IV pilus system participate in the negative regulation of swarming motility. J Bacteriol 194:5388–5403. doi:10.1128/JB.00899-12 22865844 PMC3457191

[54] Qi YH, Huang L, Liu GF, Leng M, Lu GT. 2020. PilG and PilH antagonistically control flagellum-dependent and pili-dependent motility in the phytopathogen Xanthomonas campestris pv. campestris. BMC Microbiol 20:37. doi:10.1186/s12866-020-1712-3 32070276 PMC7029496

[55] Durgadevi R, Kaleeshwari R, Swetha TK, Alexpandi R, Karutha Pandian S, Veera Ravi A. 2020. Attenuation of Proteus mirabilis colonization and swarming motility on indwelling urinary catheter by antibiofilm impregnation: an in vitro study. Colloids Surf B Biointerfaces 194:111207. doi:10.1016/j.colsurfb.2020.111207 32590245

[56] Wasfi R, Hamed SM, Amer MA, Fahmy LI. 2020. Proteus mirabilis biofilm: development and therapeutic strategies. Front Cell Infect Microbiol 10:414. doi:10.3389/fcimb.2020.00414 32923408 PMC7456845

[57] Hernandez RJ, Hesse E, Dowling AJ, Coyle NM, Feil EJ, Gaze WH, Vos M. 2019. Using the wax moth larva Galleria mellonella infection model to detect emerging bacterial pathogens. PeerJ 6:e6150. doi:10.7717/peerj.6150 30631644 PMC6322482

[58] Sheehan G, Dixon A, Kavanagh K. 2019. Utilization of Galleria mellonella larvae to characterize the development of Staphylococcus aureus infection. Microbiology (Reading) 165:863–875. doi:10.1099/mic.0.000813 31107207

[59] Germain E, Castro-Roa D, Zenkin N, Gerdes K. 2013. Molecular mechanism of bacterial persistence by HipA. Mol Cell 52:248–254. doi:10.1016/j.molcel.2013.08.045 24095282

[60] Levin-Reisman I, Balaban NQ. 2016. Edited by J. Michiels and M. Fauvart. Quantitative measurements of type I and type II persisters using ScanLag, p 75–81. Springer New York, New York, NY. 10.1007/978-1-4939-2854-5_7 26468101

[61] Carraro N, Poulin D, Burrus V. 2015. Replication and active partition of integrative and conjugative elements (ICEs) of the SXT/R391 family: the line between ICEs and conjugative plasmids is getting thinner. PLOS Genet 11:e1005298. doi:10.1371/journal.pgen.1005298 26061412 PMC4489591

[62] Gu Q, Zhu X, Yu Y, Jiang T, Pan Z, Ma J, Yao H. 2024. Type II and IV toxin-antitoxin systems coordinately stabilize the integrative and conjugative element of the ICESa2603 family conferring multiple drug resistance in Streptococcus suis. PLoS Pathog 20:e1012169. doi:10.1371/journal.ppat.1012169 38640137 PMC11062541

[63] Guédon G, Libante V, Coluzzi C, Payot S, Leblond-Bourget N. 2017. The obscure world of integrative and mobilizable elements, highly widespread elements that pirate bacterial conjugative systems. Genes (Basel) 8:337. doi:10.3390/genes8110337 29165361 PMC5704250

[64] Jaszczur MM, Pham P, Ojha D, Pham CQ, McDonald JP, Woodgate R, Goodman MF. 2024. Pathogen-encoded Rum DNA polymerase drives rapid bacterial drug resistance. Nucleic Acids Res 52:12987–13002. doi:10.1093/nar/gkae899 39413207 PMC11602152

