# Supplementary material for: Interaction of the SXT/R391 element ICEPmiJpn1 with its natural host Proteus mirabilis
Source: Microbiol Spectr. 2025 May 23;13(7):e00339-25. doi: 10.1128/spectrum.00339-25 (PMC12210918; doi:10.1128/spectrum.00339-25)

Figure S1. Growth of *P. mirabilis* in BHI, TSB, LB and PMSM media. Growth measured by OD_{600} of PmBR574-ICE, PmBR28-ICE, PmBR51-ICE and their parental strains. Mean values of three biological experiments, each with two technical replicates. Error bars represent the standard error of the mean.

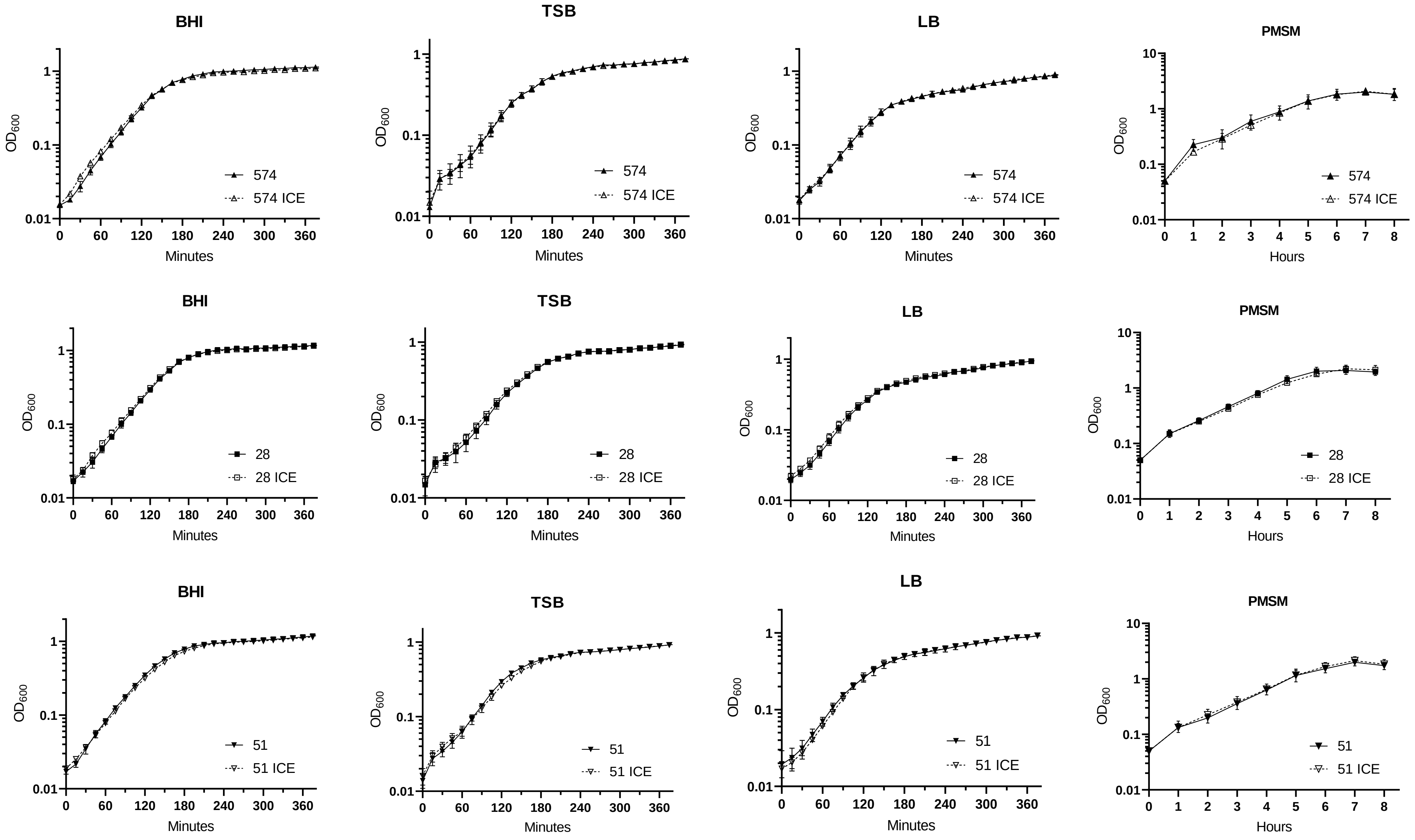

Supplement: Fig. S1 — Growth of P. mirabilis in different media. [file spectrum.00339-25-s0002.pdf]
