# Supplementary material for: Interaction of the SXT/R391 element ICEPmiJpn1 with its natural host Proteus mirabilis
Source: Microbiol Spectr. 2025 May 23;13(7):e00339-25. doi: 10.1128/spectrum.00339-25 (PMC12210918; doi:10.1128/spectrum.00339-25)

Figure S2. Self-recognition during swarming motility. Comparative assay of *P. mirabilis* strains carrying ICE*Pmi*Jpn1(PmBR574-ICE, PmBR28-ICE and PmBR51-ICE) with their parental strains (A) and with PmBR19 strain (B). Orange arrow indicates the formation of Dienes' lines.

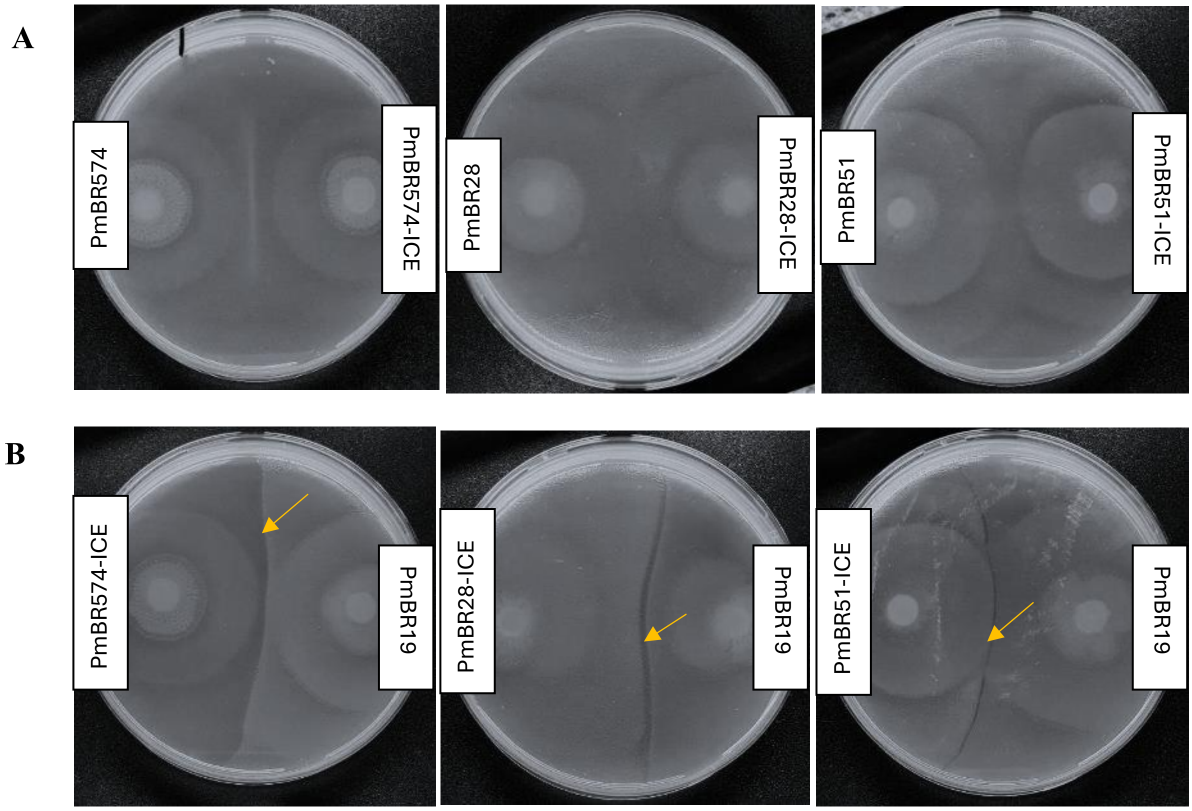

Supplement: Fig. S2 — Self-recognition of different strains during swarming motility. [file spectrum.00339-25-s0003.pdf]
