# Supplementary material for: Interaction of the SXT/R391 element ICEPmiJpn1 with its natural host Proteus mirabilis
Source: Microbiol Spectr. 2025 May 23;13(7):e00339-25. doi: 10.1128/spectrum.00339-25 (PMC12210918; doi:10.1128/spectrum.00339-25)

Figure S3. Effect of ICE*Pmi*Jpn1 on swarming motility and biofilm formation of different *P. mirabilis* strains. A: Comparison of swarming migration distances (in millimeters) between isogenic pairs. Error bars indicate the standard error of the mean from 3 independent experiments, each with 3 technical replicates. B: Comparison of biofilm biomass formation, quantified by crystal violet staining measured at OD_{550}. Error bars represent the standard error of the mean from four independent experiments, each with eight technical replicates. *P ≤ 0.05, ns: statistically not significant.

A

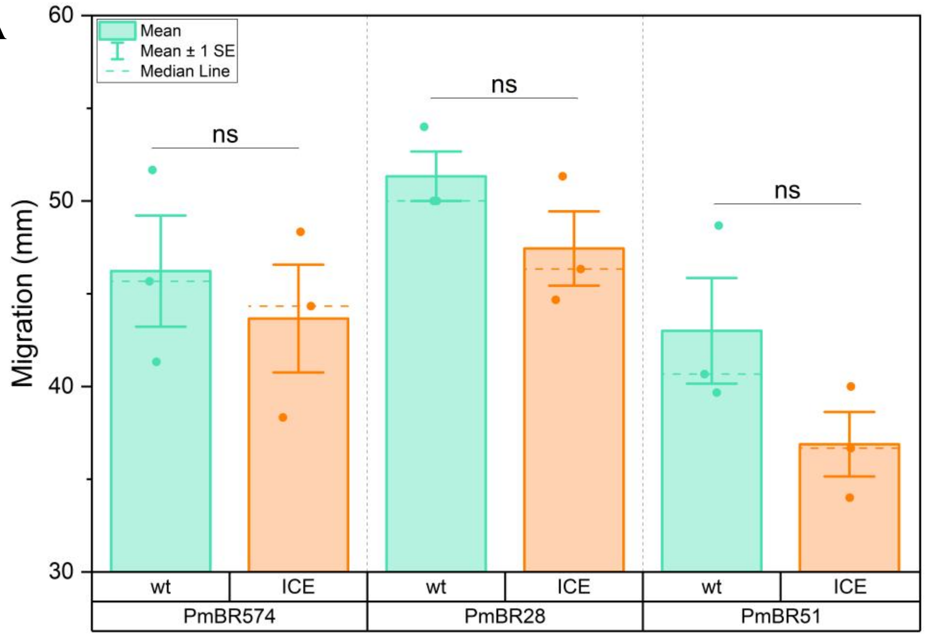

B

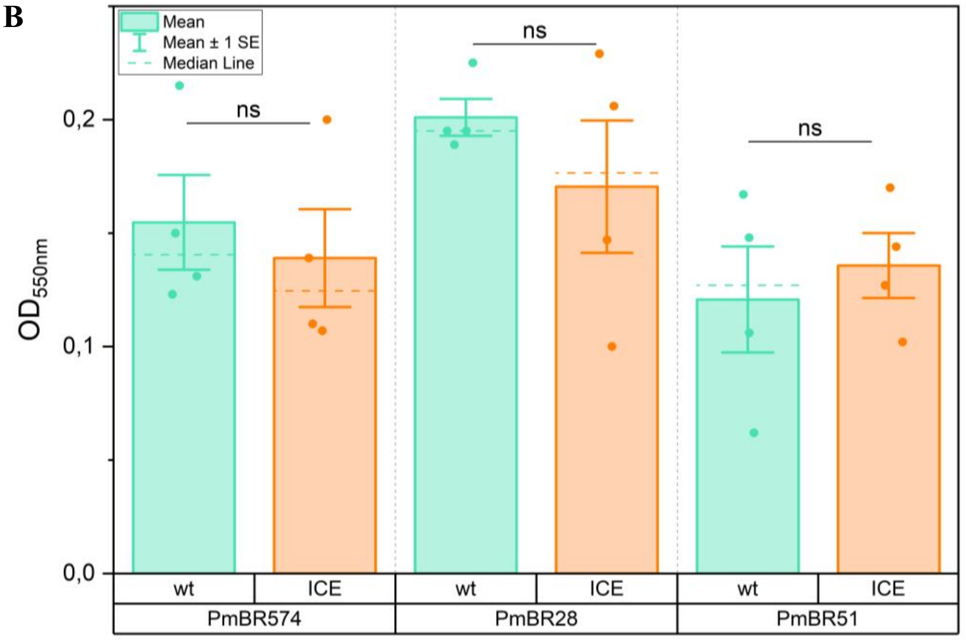

Supplement: Fig. S3 — Swarming motility and biofilm formation of different P. mirabilis strains. [file spectrum.00339-25-s0004.pdf]
