# Supplementary material for: Interaction of the SXT/R391 element ICEPmiJpn1 with its natural host Proteus mirabilis
Source: Microbiol Spectr. 2025 May 23;13(7):e00339-25. doi: 10.1128/spectrum.00339-25 (PMC12210918; doi:10.1128/spectrum.00339-25)

Figure S4. Conjugative transfer frequencies of ICE*Pmi*Jpn1 from different *P. mirabilis* donor strains to *E. coli* recipients in 3-hour mating experiments and recipient cell killing during conjugation. A: Conjugative transfer frequencies after 3h incubation. B and C: Cell counts of donors and recipients after 3h conjugation, in mating with PmBR622-ICE (B) and with PmBR28-ICE (C). Each dot represents an independent experiment, bars represent the mean and error bars represent the standard error. *P ≤ 0.05. Fold-change included in B and C.

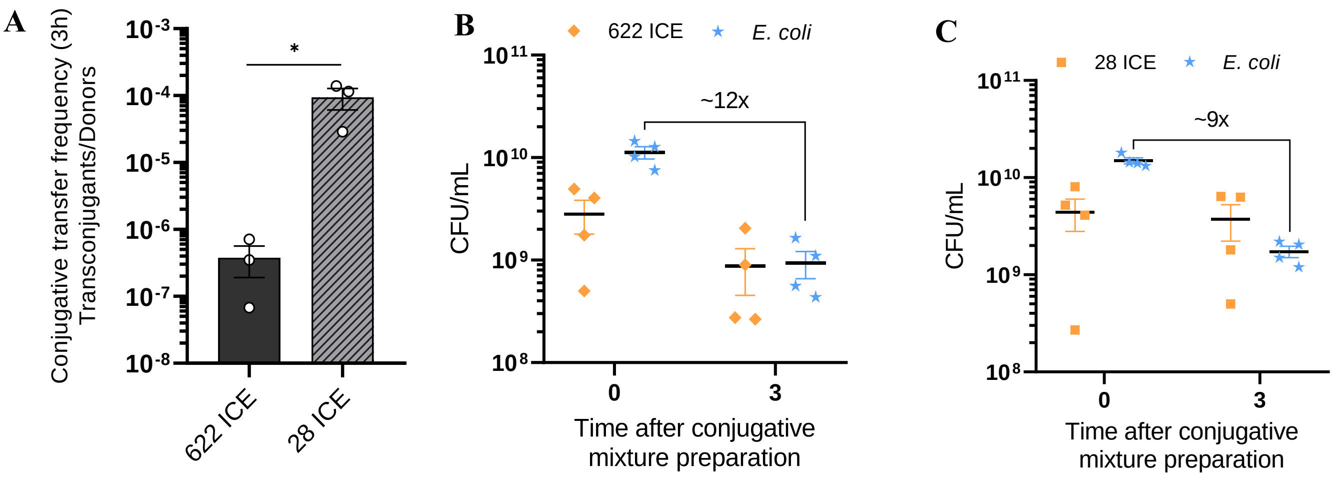

Supplement: Fig. S4 — Conjugative transfer frequencies of ICEPmiJpn1 from different P. mirabilis donor strains. [file spectrum.00339-25-s0005.pdf]
